# Impact of occupational sedentary behavior on mental health: A systematic review and meta-analysis

**DOI:** 10.1371/journal.pone.0328678

**Published:** 2025-08-20

**Authors:** Hijrah Nasir, Martine Duclos, Reza Bagheri, Alistair Cole, Julien S. Baker, David Thivel, Frederic Dutheil

**Affiliations:** 1 Université Clermont Auvergne, Laboratory of the Metabolic Adaptations to Exercise under Physiological and Pathological Conditions (AME2P), CNRS, LaPSCo, Clermont-Ferrand, France; 2 Department of Sport Medicine and Functional Exploration, Université Clermont Auvergne, INRAE, UNH, CHU Clermont-Ferrand, Clermont-Ferrand, France; 3 University of Isfahan, Exercise Physiology, Isfahan, Iran; 4 Université Lumière - Lyon 2, SciencePo Lyon, Lyon, France; 5 Hong Kong Baptist University, Centre for Health and Exercise Science Research, Kowloon Tong, Hong Kong; 6 Laboratory of the Metabolic Adaptations to Exercise under Physiological and Pathological Conditions (AME2P), Université Clermont Auvergne, Clermont-Ferrand, France; 7 Université Clermont Auvergne, CNRS, LaPSCo, Physiological and Psychosocial Stress, University Hospital of Clermont-Ferrand, Preventive and Occupational Medicine, Witty Fit, Clermont-Ferrand, France; University of Stirling, UNITED KINGDOM OF GREAT BRITAIN AND NORTHERN IRELAND

## Abstract

**Background:**

Despite numerous meta-analyses on the effects of leisure time sedentary behavior, the effect of sedentary behavior at work on mental health is largely under debate. We aimed to systematically identify and synthetize the literature examining the association between sedentary behavior at work on mental health.

**Method:**

PubMed, Embase, Cochrane, and Psycinfo databases were searched for articles reporting risks for mental health due to occupational sedentary behavior. We computed random-effects meta-analysis using all risks and both intermediate and severe levels of mental health issues, following by sensitivity analysis on severe mental health issues using 1) all risks, then only 2) fully adjusted and 3) crude or less adjusted lowest risks (pessimistic models), and 4) fully adjusted and 5) crude or less adjusted highest risks (optimistic models). We conducted meta-regression on possible influencing factors.

**Results:**

We included 12 studies in the systematic review and 7 in the meta-analysis, for a total of 40,314 workers (35 years old, 65.5% men). Exposure to sedentary behavior at work increased the risk of intermediate and severe mental health issues by +34% (95 CI 18–49%). All sensitivity analyses on severe mental health issues were also significant, whatever the model: + 35% (12–58%) using all risks, 39% (15–63%) using fully adjusted pessimistic model, + 36% (13–59%) using crude or less adjusted pessimistic model, 85% (27–143%) using fully adjusted optimistic model, + 85% (26–143%) using crude or less adjusted optimistic model. Age may have an increased risk of mental health issue when exposed to occupational sedentary behavior, while high education reduced the risk.

**Conclusion:**

Our meta-analysis shows that occupational sedentary behavior increases the risk of mental health issue. Inconsistent results precluded robust conclusion for variables that may further influence this risk.

## Introduction

Modern society is associated with a switch from workers employed in goods producing and agricultural sector into service occupations that are mostly sedentary [1]. Nowadays, most adults spend their time being sedentary, in leisure time such as watching TV [2], but also mainly at work [3], for a total of around 10 hours of sedentary time per day [4,5]. Sedentary behavior is defined as any waking behavior characterised by an energy expenditure <1.5 Metabolic equivalent of task (METS) while in a sitting or reclining posture [6]. Many research linked sedentary behavior with poorer health-related outcomes, particularly overweight and obesity [7], cardiovascular diseases and type 2 diabetes [8,9], and all-cause mortality [10,11]. Besides, sedentary behavior was also linked to mental health issues. Leisure time sedentary behavior is a risk factor for poorer mental health [12–15]. Several meta-analysis demonstrated that individuals with higher sedentary behavior had a significantly higher risk of depression compared to those who reported being less sedentary [16–19]. However, those meta-analysis did not focus specifically on the effect of occupational sedentary behavior. To our knowledge, no meta-analysis has assessed the relation between sedentary behavior at work and mental health. Moreover, common associated risk factors of poorer mental health such as female gender [20,21], single [22,23], obesity [24], lower education [25], or physical activity level [26] were poorly evaluated in the context of occupational sedentary behavior. In more details, older adults have an increased risk of cognitive and mood disorders, including dementia and depression, with an estimated 15% of the older people suffered from depressive symptoms [27]. Female sex was reported to be a significant predictor of depression, anxiety, as well as stress [28–30]. Some studies investigated that higher BMI is associated with impaired mental health as well as a range of cognitive factors [31], depression [32] and anxiety [33]. Higher education attainment was associated with a lower risk of anxiety and depression [33,34], psychological distress [35], and psychiatric disorders [36]. Furthermore, unmarried individuals may face higher risks of depression [37], perceived stress and anxiety [38] compared to married individuals. Similarly, low physical activity was detrimentally linked to depression and anxiety [33,39,40] and leisure-time physical activity regardless the intensity was associated with lower incidence of future depression [41]. However, those studies did not take into account sedentary behavior as a risk factor, and the association between occupational sedentary behavior and mental health issues taking into account some sociodemographic factors was not widely discussed. Assessing who is the most at risk of poorer mental health when exposed to sedentary behavior at work is particularly important to build efficient preventive strategy.

Therefore, we aimed to conduct a systematic review and meta-analysis to study the impact of sedentary behavior at work on mental health. We also aimed to compare the risk on mental health depending on the types of mental pathologies and putative influence of sociodemographic factors (i.e., sex, body mass index (BMI), education, marital status).

## Materials and methods

### Literature search

We reviewed all studies reporting the impact of occupational sedentary behavior on mental health. The search was conducted on the PubMed, Embase, Cochrane, and Psycinfo databases with the following keywords: “sedentary behavior” and (“mental health” or stress or depression or anxiety) and (occupation or work). The search was conducted up to August 2024 (details for the search strategy used within each database are available in S1 File).

### Eligibility (inclusion and exclusion) criteria

To be included in the systematic review, studies had to be peer-reviewed and to report both data on occupational sedentary behavior and on mental health in workers. More specifically, articles had to report either sedentary time at work or total sedentary behavior (including sedentary time at work). When a risk (relative risk, hazard ratio, or odds ratio) of mental health issues was described in workers exposed to occupational sedentary behavior, studies were further included in the meta-analysis. We excluded population other than adult workers, in a normal range of age – thus we excluded putative studies on children as well as studies on elderly. We also excluded articles not written in English. Furthermore, reference lists of publications meeting the inclusion criteria were manually searched to identify possible further articles not retrieved from electronic search with our keywords.

### Search strategies and selection process

Two authors (Hijrah Nasir and Reza Bagheri) conducted the literature searches, reviewed the abstracts, decided the suitability of the articles for inclusion and extracted the data, and selected the included papers. A third author (Frédéric Dutheil) reviewed the articles where consensus on suitability was debated. Finally, all authors reviewed eligible articles. We followed the Preferred Reporting Items for Systematic Reviews and Meta-Analyses (PRISMA) guidelines (S1 Table) [42].

### Data extraction

The collected data consist of author’s name, publication year, title, study design, country, objectives and outcomes of included articles, inclusion and exclusion criteria, sample size, main characteristics of individuals (age, sex, education, occupation, BMI, physical activity level, and marital status), level of sedentary exposure (as reported in articles, i.e., higher than a cut-off of hours of sitting per day or per week chosen by authors), characteristics of mental health issues (type, i.e., psychological distress, stress, anxiety, depression, deep sadness, burn out syndrome; and intensity of symptoms, i.e., low, intermediate, severe), and risk of mental health (all risks were reported with the following characteristics: crude or adjusted, variables used for adjustment, and type of risk – hazard ratio, odds ratio, relative risk, coefficient beta). Data extraction was conducted using Excel.

### Risk of bias and certainty of evidence

In order to verify the quality of the study, we used the Scottish Intercollegiate Guidelines Network (SIGN) checklist for cohort studies to evaluate the main causes of bias through 14 items with four possible answers (yes, no, can’t say or not applicable) [43]. Furthermore, we used the Newcastle-Ottawa Scale (NOS) to evaluate the risk of bias of included observational studies [44]. The NOS consists of three dimensions – selection, comparability, and outcome – for a total of nine items scored one point each and providing a maximum score of 9 (S2 File) [44]. Scores at the SIGN and NOS checklist were converted into a percentage (one point was given per criteria fulfilled, divided by the sum of applicable criteria). Besides, we also used the Grading of Recommendations Assessment, Development and Evaluation (GRADE) guidelines as the evidence synthesis for the review (S2 Table) [45].

### Statistical analysis

The statistical analyses were conducted using STATA® software (v15, StataCorp, College Station, USA). Quantitative variables were described as mean and standard deviation, and qualitative (categorical) variables were presented in the form of number and associated percentage. We computed random effects meta-analyses (DerSimonian and Laird approach) when data could be pooled to calculate the risk of mental health issues in workers exposed to occupational sedentary behavior [46]. We stratified meta-analyses on type of mental health issues, i.e., psychological distress, stress, anxiety and/or depression, deep sadness, and burn out syndrome. We described our results by calculating the hazard ratio, i.e., the risk of mental health issues for workers exposed to occupational sedentary behavior – either sedentary time at work or total sedentary behavior (including sedentary time at work) –, and its associated 95% confidence intervals (95 CI). Hazard ratio were centered at one if the prevalence of mental health issues in workers exposed to occupational sedentary behavior did not differ from the prevalence of mental health issues in workers not exposed to occupational sedentary behavior. Hazard Ratio >1 with the lower limit of 95 CI also >1 denoted an increased risk of mental health issues. Hazard Ratio <1 with the upper limit of 95 CI also <1 reflected a decreased risk of mental health issues for workers with occupational sedentary behavior. We computed a main meta-analysis based on the risk of both intermediate and severe mental health issues, and taking into account all risks mentioned in each study (crude and adjusted). Then, we computed five sensitivity meta-analyses. First, we repeated aforementioned meta-analysis with all risk listed in all studies (crude and all adjusted risks) but we took into account only the highest level of symptoms for each included study (severe mental health issues). Then, to preclude a bias of selection of data, we used a method that we previously published and computed four meta-analyses with the inclusion of only one risk per study (crude/less adjusted or fully/most adjusted, and pessimistic, i.e., the lowest risk or optimistic model, i.e., the highest risk) [47]. Therefore, the six meta-analyses were: all risks of intermediate + severe mental health issues, all risks of severe mental health issues, crude/ pessimistic risks, fully adjusted/ pessimistic risks, crude/ optimistic risks, and fully adjusted/ optimistic risks. We further repeated again those six meta-analyses using only the studies that specifically assessed a risk associated with occupational sitting time (sensitivity analyses). Heterogeneity between studies was evaluated by examining forest plots, 95% confidence intervals (95 CI) and I-squared (I²). I^2^ is a measure of heterogeneity that ranges from 0 to 100%. Heterogeneity is low under 25%, modest between 25 and 50%, and high above 50%. In order to verify the strength of our results, we repeated aforementioned meta-analyses after exclusion of outliers from meta-funnels. Finally, when we get enough data (>5 studies), we computed meta-regressions to compare the risk between each type of mental health issue (psychological distress, stress, anxiety, depression, deep sadness, vs burnout syndrome) and to identify the association between the risk of mental health issue and sociodemographic (age, sex, body mass index – BMI, marital status, education) and lifestyle behavior (physical activity). The results were presented in 95% confidence intervals (95 CI) and corresponding p-values. Statistical significance was marked by p-value ≤0.05.

## Results

**Fig 1:**
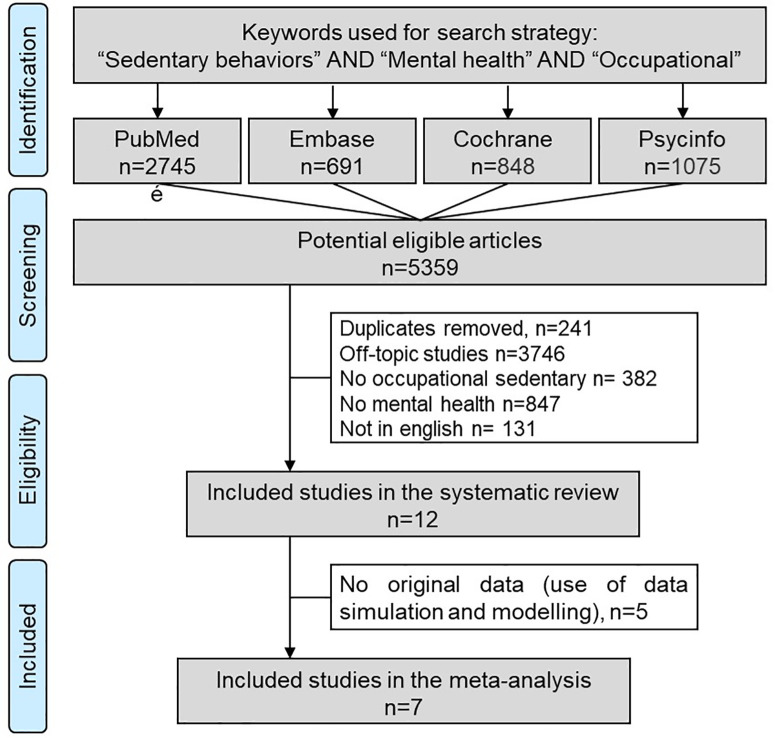
Search strategy. We followed the Preferred Reporting Items for Systematic Reviews and Meta-Analyses (PRISMA) guidelines for the search strategy.

An initial search produced a possible of 5,359 articles. After removal of duplicates and use of the selection criteria, we included 12 articles in the systematic review reporting the impact of occupational sedentary behavior on mental health, from which 7 articles reporting a risk of mental health issues when exposed to occupational sedentary behavior were included in the meta-analysis (Fig 1). All articles included were written in English. Main characteristics of studies included in the systematic review are presented in Table 1 and S1 Dataset, and characteristics of studies included in the meta-analysis are presented in Table 2 and S1 Dataset. We describe below only the articles included in the meta-analysis, except in the section narrative review that is dedicated to the articles included only in the systematic review and not in the meta-analysis.

**Table 1:** Characteristics of studies included in the systematic review but not in the meta-analysis.

Study	Country	Design	Characteristics of workers	Type of statistics	Main objective of the study	Main results of the study	Mental health issue	Results related to mental health and sedentary behavior	Reason for non-inclusion in the meta-analysis
**Hanna 2019**	Qatar	Cross-sectional study	n = 479 43% male, University employee	OR	To assess the association between levels of sedentarybehavior, physical activity, back pain and psychosocial factor in university employees	Sedentary behavior was significantly associated with low back pain and upper back pain (OR=1.74, 95 CI 1.19 to 2.57)	Depressed mood	The risk of “sitting too much” was increased in workers with depressed mood (OR=1.41, 95 CI 0.77 to 2.63, P = 0.056)	Unmatched risk (risk of long sedentary behavior for workers with a depressed mood)
**Muniswamy** **2021**	India	Randomized controlled trial	n = 2,476 26.3 ± 3.5 yo, 65% male, IT workers	Estimated mean ± SE F P ŋ2	To assess the short-termeffects of a social media-based intervention on thephysical and mental health in desk-based remote office workers during the pandemic	Physical and mental health improved after a short-term social media intervention in desk-based remote office workers	Stress, anxiety, and depression	The intervention group reduced occupational sitting time by 58 min per working hours, and decreased their stress and anxiety scores by 6 points and 3 points (using DASS21 questionnaire) compared with controls	No risk (changes in scores of stress and anxiety)
**Sakakibara** **2023**	Japan	Cross-sectional study	n = 1,213, (white collars n = 1077, 41.8 ± 10.3 yo, 47.8% male; blue-collars n = 1036, 40.8 ± 10.6 yo, 85.3% male)	Estimated mean ± SE F P ŋ2	To investigate the association between occupational sedentary behavior and mental health and work engagement among white- and blue-collar workers	White-collar workers with high sedentary behavior at work had a significant poorer mental health and a lower work engagement compared to those with low sedentary behavior, without difference between low and high sedentary blue-collar workers	Mental health	High sedentary behavior was associated with poorer mental health (in adjusted model included sociodemographic, work-related variables, and MVPA) in white-collar workers but not in blue-collar workers	No risk
**Sung 2021**	South Korea	Cross-sectional study	n = 6,359 40-70 yo, blue, pink, and white-collars	OR	To assess the association of occupation with MVPA, MSA and sedentary behavior in middle-aged workers	White-collar workers had higher sedentary behavior than blue-collars. -MSA participation was lower in blue-collar workers; MVPA and MSA were negatively associated with long working hours	Stress perception	The risk of sedentary behavior increased in workers with high stress compared to those with low stress (men: OR=1.36, 95 CI 1.13 to 1.57; women: OR=1.13, 95 CI 0.96 to 1.34)	Unmatched risk (risk of high sedentary behavior for workers with high stress)
**Xu 2023**	UK	Prospective cohort	175,543 52.6 ± 7.1 yo, 50.3% male, 16.2% shift workers	HR	To explore the associations between shift work, and anxiety and depression	Shift workers had a higher risk of depression and anxiety	Depression and anxiety	Shift workers was associated with a higher risk of depression (HR = 1.22, 95 CI 1.12 to 1.33, P < .001) and anxiety (HR = 1.16, 95 CI 1.04 to 1.28, P < 0.001) compared to non-shift workers by taking into account smoking, sedentary time, BMI, and sleep duration as variables	Unmatched risk (no data about occupational sedentary behavior)

*HR: hazard ratio, MSA:muscle-strengthening activities, MVPA: moderate to vigorous physical activity, OR: odds ratio, SE: standard error*.

**Table 2:** Characteristics of studies included in the meta-analysis (articles using original data and describing either an odds ratio, a relative risk, or a hazard ratio, or giving data to calculate this risk).

Study	Country	Time Period	Design	Population				Mental health			Sedentary behavior	
				Total n	Men, %	Age, year	Occupation	Type	Measured by	Adjustment	Exposure	Measured by
**Hagger-Johnson 2014**	UK	1997/99 1997/99–2002/04 1997/99–2007/09	Cross-sectional study and Prospective cohort	11,592	67	35-55	Civil servants	Psychological distress	General Health Questionnaire (GHQ-30); the Short Form-36 Mental Component Summary (SF-36 MCS); Centre for Epidemiologic Studies Depression Scale (CES-D)	Age, sex, socio-economic status, education, physical activity, marital status, retirement, smoking status, alcohol use, diet, chronic disease, BMI, C-reactive protein, interleukin-6	Medium (sitting a total of 33–50 h/week) High (sitting a total of 51–135 h/week)	Self-Administered Physical Activity Questionnaire
**Kilpatrick 2013**	Australia	2010	Cross-sectional study	3,367	28.1	Age category	Government employees	Psychological distress	Kessler Psychological Distress scale (K10)	Age, marital status, effort-reward imbalance, and leisure-time physical activity	Moderate sitting:3–6 h/day High sitting: > 6 h/day	International Physical Activity Questionnaire (IPAQ)
**Hallgren 2020**	Sweden	Jan 2017- Jun 2019	Cross-sectional study	23,644	57	42.5 ± 12.0	Targeted employees in the companies	Depression, anxiety	Questionnaire: ‘I experience worry, depressed mood or anxiety...’ with 5 response alternatives; Very often, Often, Sometimes, Rarely, and Never	Age, gender, education, smoking, pain, medication, BMI, exercise frequency	25%–49% of time 50%–74% of time ≥75% of time Almost always sedentary	Swedish Health Profile Assessment (HPA) survey
**Martín-del-Campo 2023**	Mexico	Apr-Aug 2020	Retrospective cohort	1,146	38	37 ± 8	Workers in tertiary hospital	Stress, anxiety, depression	The Spanish short version of the Depression, Anxiety and Stress Scales (DAAS-21)	Age, sex, marital status, work category, diabetes, hypertension, pulmonary disease, obesity (BMI > 40 kg/m2), assignment to COVID-19 areas, frequency of walking/ intense and moderate physical activity/ sedentary behavior, presence of depression, anxiety or stress	>6 h sitting/workday	Spanish short form of the International Physical Activity Questionnaire (IPAQ)
**Muniswamy 2022**	India	March–Jun 2021	Cross-sectional study	80	65	25.7 ± 3.1	IT workers	Stress, anxiety, depression	Depression Anxiety Stress Scale (DASS 21)	Age, gender, and BMI	>6 h sitting/workday	Modified workers’ living activity time questionnaire (WLAT-Q)
**Nascimento 2020**	Brazil	2018	Cross-sectional study	254	83.5	Age category	Police officers	Deep sadness, burnout syndrome	EPS-10 stress perception scale, Youth Risk Behavior Surveillance System (YRBSS) for suicidal ideation, HADS, Maslach Burnout Inventory	Age, gender, BMI, work type, weekly workload, workplace, service time, PA level	“Low levels of physical activity = 1” was given for “insufficiently active” and “sedentary”	The short version of the International Physical Activity Questionnaire (IPAQ)
**Watanabe 2021**	Japan	Feb 2019 – March 2020	Prospective cohort	231	75.8	Age category	Targeted employees in the companies	Major depression episode	Composite International Diagnostic Interview version 3.0 (CIDI 3.0)	Age, sex, physical activity levels, educational status, marital status, household income, job stressors (job demands, job control, supervisor, and co-worker support), and working hours	9.5 + h/day (vs < 9.5 h/day)	The Worker’s Living Activity-time Questionnaire (JNIOSH- WLAQ)

*BMI: body mass index, SB: sedentary behavior, PA: physical activity, HADS: Hospital Anxiety and Depression Scale*.

### Risk of bias and certainty of evidence

**Fig 2:**
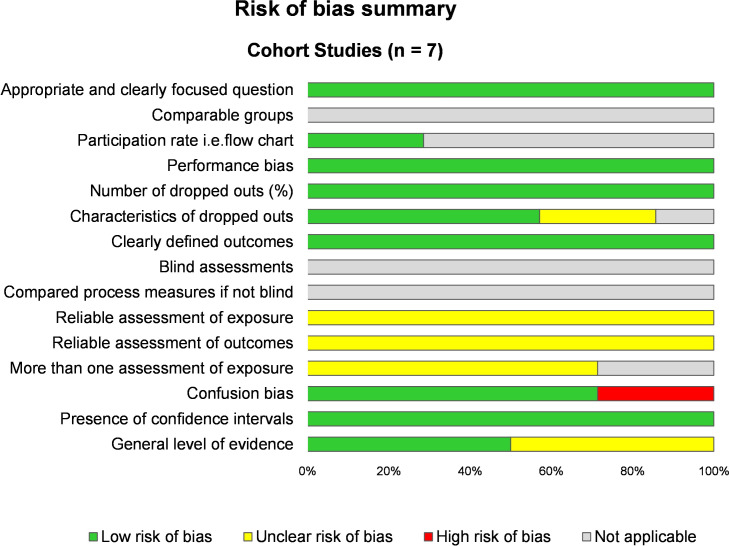
Risk of bias summary using the Scottish Intercollegiate Guidelines Network (SIGN) checklist. We used the Scottish Intercollegiate Guidelines Network (SIGN) grid for cohort studies that consist of 14 items, evaluating the main causes of bias through 4 possible answers (yes, no, can’t say or not applicable).

According to SIGN cohort checklist, the methodological quality of the included studies was good with a mean score of 68.3 ± 8.9%, ranging from 58.3% [5,48] to 78.6% [49]. There was an unclear risk of bias for the assessment of outcomes and exposures, as well as for confusion bias (Fig 2). Using the NOS checklist, the overall quality of studies was 66.7 ± 11.9%, with three studies had a score greater than two third [5,20,49]. All included articles mentioned ethical approval, except one study [50]. According to GRADE checklist, the reliability of evidence was scored high for our main outcome and moderate for most secondary outcomes (S2 Table).

### Aims and outcomes of included studies

The main objective of all included studies was to examine the associations between occupational sitting and mental health in employed adults, independent of leisure-time physical activity, except one study that had for main objective to examine the associations between leisure and occupational sitting and mental health [12].

### Study designs

Four studies were cross-sectional [12,21,48,50], one was a prospective cohort [49], one study used both a cross sectional and prospective design [5], and one was retrospective [20]. These studies were located in Asia (Japan [49], and India [21]), Australia [50], Europe (Sweden [12] and UK [5]), and South America (Brazil [48] and Mexico [20]).

### Recruitment, inclusion, and exclusion criteria

All studies recruited workers from targeted population. Four studies recruited workers from public sectors: government agency [5,50] and military police officers [48] and health-care workers [20]; and three studies recruited workers from private companies: healthcare services [12], remote working information and technology [21], and manufacturing and transportation company [49]. Age was an inclusion criteria in five studies: > 18 years [20], > 20 years [49], age 23–35 years [21], age 21–56 years [48], age 35–55 years [5]. Some studies added the following inclusion criteria: comprehension of Japanese [49], having >1 year of experience in the IT industry and a similar designation in the company for the past 1 year, with a Facebook account and at least login once in 2 days [21]. The two other studies did not clearly define the inclusion criteria [48,50]. Only three studies reported exclusion criteria [21,48,49]. Two of them excluded workers with major depressive episode or being officially absent due to mental health problems in the last 12 months [49], and having an alteration of mental or psychological health [21]. One study also excluded workers with a chronic disease or taking drugs, pregnant or who delivered in the past month, and those who planned to shift the job in the next two months [21]. One study excluded workers who took medical leave [48].

### Population

**Sample size** ranged from 80 [21] to 23,644 [12]. A total 40,314 individuals were included in the study.

**Age** was on average 35 years old (95 CI 24.0 to 46.1 years old), ranging from 25.7 [21] to 47 years old [50] using the mean age reported in four studies [12,20,21,50]. Two studies reported age category [48,49], one study reported mean age for men and women [50], and one study reported a range (min-max) [5].

**Gender** was reported in all studies. The mean proportion of men was 65.5% (95 CI 55.0 to 76.0) ranging from 38.2 [20] to 83.5% [48].

**Education** was reported in four studies [12,21,49,50]. The mean proportion of people with education higher than high school were 67.0% (95 CI 30.0 to 100.4%), ranging from 52.0% [50] to 100% [21].

**BMI** was reported in five studies [12,20,21,48,50] using category: overweight (25–30 kg/m^2^) and obese (>30 kg/m^2^) [12,21,50], all workers with a BMI > 25 kg/m^2^ [48] or >40 kg/m^2^ [20]. Including the three studies with the same cut-off [12,21,50], the percentage of obese people (>30 kg/m^2^) was 23% (14–33%), ranging from 6 [20] to 65% [48]. Two studies also reported the average BMI [21,50].

**Marital status** was reported in four studies [20,21,49,50]. Proportion of married participants was 56% (34–78%), ranging from 19 [21] to 81% [50].

**Physical activity** was auto-reported in four studies [12,21,49,50] through questionnaires. Three studies reported prevalence, i.e., percentage of workers meeting daily physical activity recommendations [21], doing never/ sometimes/ one-two/ 3–5/ or ≥6 times per week [12], or doing low, moderate, or high intensity of physical activity [49]. One study reported the average leisure time physical activity [50]. However, in our analysis, we did not take physical activity as an influencing variable due to insufficient data.

**Other descriptive variables** were seldomly reported precluding further analyses, i.e., middle or upper class [48], occupation [20], permanent or temporary contract [20], full- or part-time [50], seniority [48], shiftwork [20], working hours [5,21,48,49], telework [21], psychosocial risk factors [49,50], smoking [12,49], alcohol [49], personal medical history (such as pulmonary disease, hypertension, diabetes) [20], use of pain medication [12], and sleep duration [21].

### Instruments used to assess sedentary behavior at work

Four studies collected data on sedentary behavior using The International Physical Activity Questionnaire-Short Form (IPAQ) [20,48,50], two studies used Workers’ Living Activity Time Questionnaire (WLAT-Q) [21,49], one study used Self-Administered Physical Activity Questionnaire [5], and one study used Swedish Health Profile Assessment (HPA) survey [5]. All studies used different cut-off for sedentary behavior at work. One study defined “high sedentary” if sitting >8 h/day [33]. Two studies defined “high sedentary” if sitting >6 h/day [20,21]. One study categorized sedentary behavior into low (<3 h/day), moderate (3–6 h/day) and high sitting (>6 h/day) [50]. One study defined “high sedentary” if sitting >9.5 h/day [49]. One study categorized total sitting into “low” (<33 h/week), “medium” (33–50 h/week) and “high” sitting (51–135 h/week) [5]. One study used the percentage of sitting that were categorized into <25%, 25–49%, 50–74%, ≥ 75% of time, and almost always sedentary [5]. One study only defined sedentary behavior as insufficiently active or sedentary [48]. In our meta-analysis, when a study reported more than two categories of sedentary behavior at work, we used the risks corresponding to the highest level of sedentary behavior at work.

### Instruments used to assess mental health

Mental health data was evaluated using questionnaires. Two studies [20,21] used the *Depression Anxiety Stress Scale - 21 (DASS21)*. One study [5] used the General Health Questionnaire (GHQ-30), the *Short Form-36 Mental Component Summary (SF-36 MCS)*, as well as the *Center for Epidemiologic Studies Depression Scale (CES-D)*. One study [50] used the Kessler Psychological Distress scale (K10). One study used a simple question: *‘I experience worry, depressed mood or anxiety...’* with five possibilities of answer (very often, often, sometimes, rarely, and never) [12]. One study [48] used the *Perceived stress scale (PSS-10)*, the *Youth Risk Behavior Surveillance System (YRBSS)* for suicidal ideation, the *Hospital and Anxiety Depression Scale (HADS)*, as well as the *Maslach Burnout Inventory (MBI)*. One study used the *Composite International Diagnostic Interview version 3.0 (CIDI 3.0)* [49]. Among all those assessments, included studies only calculated a risk for psychological distress [5,50], depression [12,20,21,49], anxiety [12,20,21], stress [20,21], deep sadness [48], and burnout [48].

### Narrative synthesis on studies included only in the systematic review

Sakakibara et al. [51] demonstrated an association between higher occupational sedentary behavior and poorer mental health in blue and white-collars, but there were no risk nor data to calculate a risk for mental health issues. Hanna et al. found that the risk of “sitting too much” was increased in workers with depressed mood [52]. Similarly, Sung et al. showed that the risk of sedentary behavior increased in workers with high stress compared to those with low stress [53]. But both studies did not assess the risk in an opposite way, i.e., the risk of mental health issues in sedentary workers compared to less-sedentary workers. Muniswamy et al. showed the positive effects of a social media-based intervention on workers, by reducing their occupational sedentary behavior and their level of stress and anxiety [54]. Xu et al. showed that shift workers had a higher risk of depression and anxiety compared to non-shift workers by taking into account sedentary time [55]. Those studies did not assess the risk of occupational sedentary behavior on mental health.

### Meta-analysis on the risk of mental health

**Fig 3:**
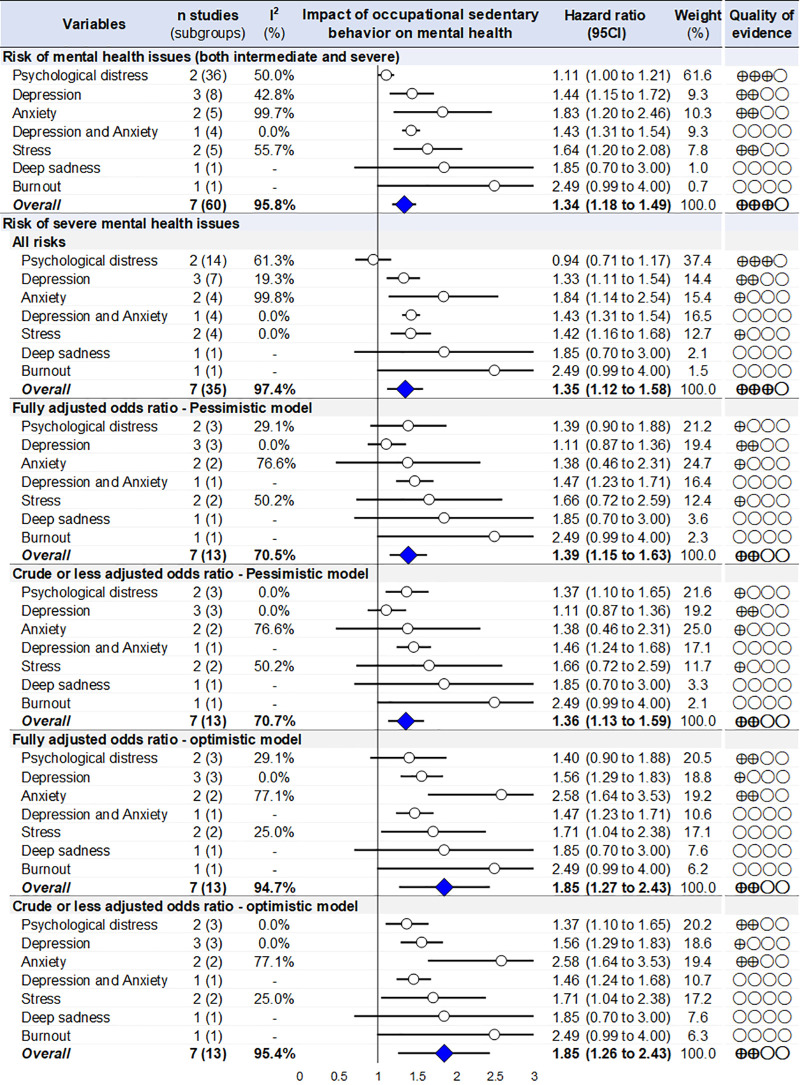
Summary of meta-analyses on the risk of mental health issues due to occupational sedentary behavior (only occupational in 4 studies and total, i.e., occupational and leisure in 3 studies). *Blue lozenges represent the overall risk of mental health after exposure to occupational sedentary behavior. Grey lozenges represent risk for each type of mental health issue after exposure to occupational sedentary behavior. The length of each horizontal line around the blue/grey lozenges represents their 95% confidence interval (95 CI). The black solid vertical line represents the null risk for mental health (with a value of 1). When horizontal lines cross the null vertical line, the risk for mental health is not significant.* I-squared (%): percentage of heterogeneity between studies for each meta-analysis; Weight (%): Weight of each study for each meta-analysis.

Using the global model with all risks both for intermediate and severe levels of mental health issues, we found that occupational (only occupational in 4 studies and total, i.e., occupational and leisure in 3 studies) sedentary behaviour was associated with an increased the risk of mental health issues by 34% (OR=1.34, 95 CI 1.18 to 1.49). Stratification by type of mental health issues showed an increased risks for all mental health issues that were reported in several studies. Due to sedentary behavior, the risk increased by 11% (1.11, 1.00 to 1.21) for psychological distress, 44% (1.44, 1.15 to 1.72) for depression, 83% (1.83, 1.20 to 2.46) for anxiety, 43% (1.43, 1.31 to 1.54) for depression and anxiety, and 64% (1.64, 1.20 to 2.08) for stress. Mental health issues reported in only study were not significant, i.e., for deep sadness (1.85, 0.70 to 3.00) despite being close to significance for burnout (2.49, 0.99 to 4.00) (Figs 3 and S1).

### Sensitivity analyses by type of risks

All sensitivity analyses on severe mental health issues were also significant, whatever the model: + 35% (12–58%) using all risks, 39% (15–63%) using fully adjusted pessimistic model, + 36% (13–59%) using crude or less adjusted pessimistic model, 85% (27–143%) using fully adjusted optimistic model, + 85% (26–143%) using crude or less adjusted optimistic model. Sensitivity analyses stratified by type of mental health issues also showed similar results, with the same types of mental health issues being significant in all models, i.e., depression, anxiety, depression and anxiety, and stress – except in the pessimistic models where depression, anxiety, and stress were no longer or less significant (Figs 3 and S1). The overall risks of mental health issue are marked with a high heterogeneity in all aforementioned meta-analysis, ranging from 71 to 97% (Figs 3 and S1). The high heterogeneity is also shown by the numerous studies that were outside the base of the meta-funnel, hindering further sensitivity analysis (S2 Fig).

### Sensitivity analyses using only occupational sedentary behavior

**Fig 4:**
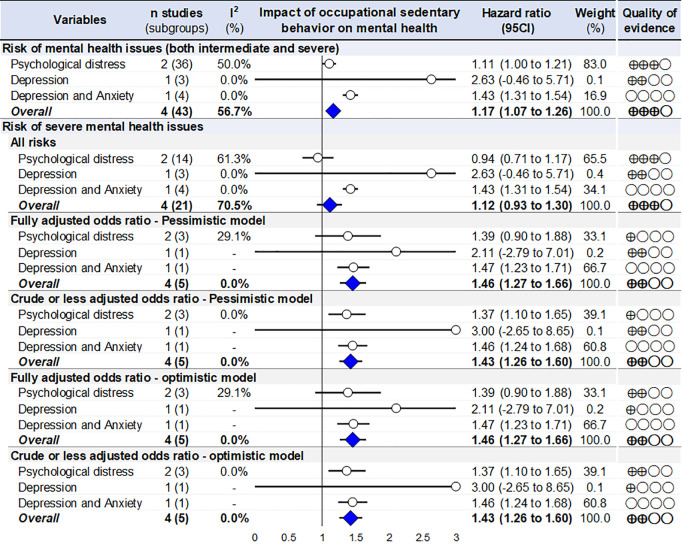
Summary of meta-analyses on the risk of mental health issues due to occupational sedentary behavior, using only the four studies that specifically assessed a risk associated with occupational sitting time. Blue lozenges represent the overall risk of mental health after exposure to occupational sedentary behavior. Grey lozenges represent risk for each type of mental health issue after exposure to occupational sedentary behavior. The length of each horizontal line around the blue/grey lozenges represents their 95% confidence interval (95 CI). The black solid vertical line represents the null risk for mental health (with a value of 1). When horizontal lines cross the null vertical line, the risk for mental health is not significant. I-squared (%): percentage of heterogeneity between studies for each meta-analysis; Weight (%): Weight of each study for each meta-analysis.

Repetition of all aforementioned meta-analyses with only the four studies that specifically assessed a risk associated with occupational sitting time, all results remained significant. Occupational sedentary behavior was associated with an increased risk of mental health issues using the global model with all risks both for intermediate and severe levels of mental health issues (+17% of risk; OR=1.17, 95 CI 1.07 to 1.26), as well as for meta-analyses on severe mental health issues: 46% (27–66%) using fully adjusted pessimistic model, + 43% (26–60%) using crude or less adjusted pessimistic model, 46% (27–66%) using fully adjusted optimistic model, + 43% (26–60%) using crude or less adjusted optimistic model, but not anymore significant for model taking all risk of severe mental health: + 12% (−10% to +30%) (Fig 4).

### Meta-regressions, i.e., putative influencing factors

We only computed meta-regressions on meta-analyses with the seven studies (insufficient data otherwise). Considering the type of mental health issues, the risk is higher for anxiety in the crude optimistic model (versus psychological distress and depression), and the risk is higher for anxiety than psychological distress in the two models with all risks.

**Fig 5:**
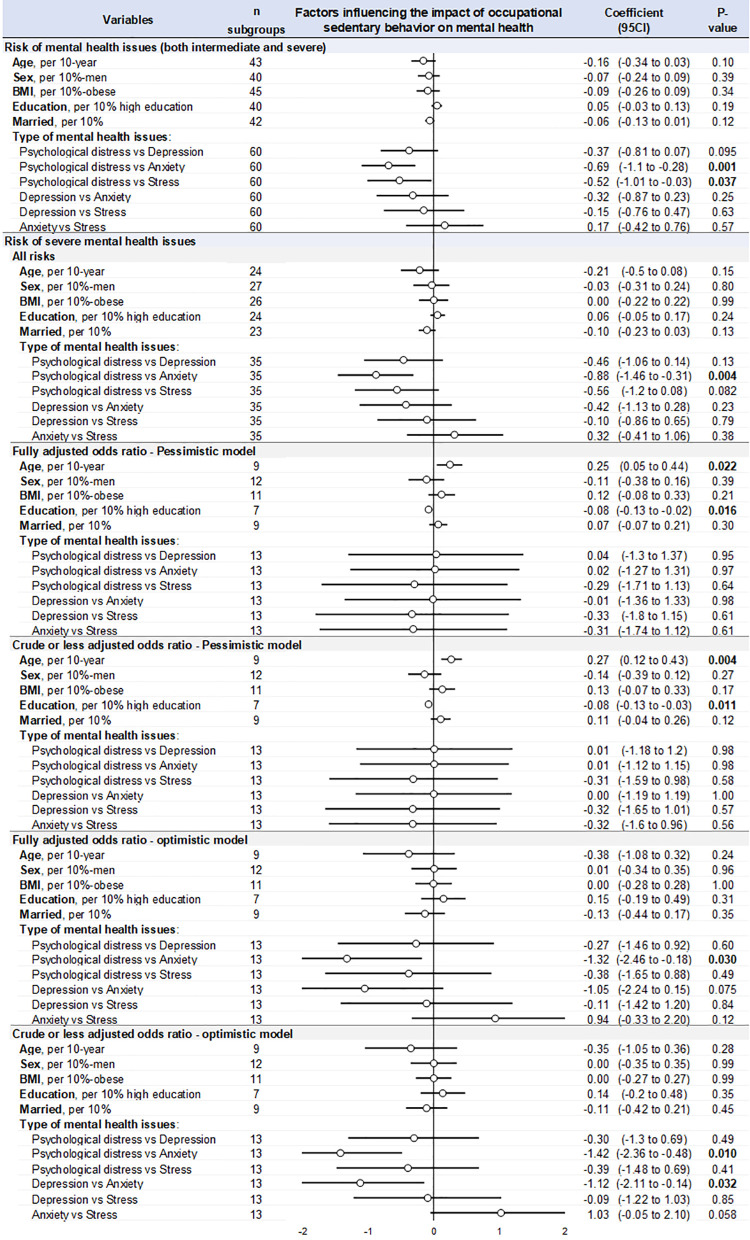
Meta-regressions, i.e., variables influencing the risk of mental health issues, depending on groups: 1) risk of mental health (both intermediate and severe), 2) risk of severe mental health (all risks), 3) risk of severe mental health pessimistic model (crude and fully adjusted), 4) risk of severe mental health optimistic model (crude and fully adjusted). The effect of each variable on the risk of mental health issues is represented by a dot on a horizontal line in the forest-plot. The dots represent the coefficient for each variable, and the length of each line around the dots represent their 95% confidence interval (95 CI). The black solid vertical line represents the null estimate (with a value of 0). Horizontal lines that cross the null vertical line represent non-significant variables on the risk of mental health issues.

In the pessimistic models, when exposed to occupational sedentary behavior, older age increased the risk of severe mental health issues by 25% (95 CI 5–44%) per 10-year for fully adjusted odds ratio and by 27% (12–43%) per 10-year for crude odds ratio. On the opposite, higher level of education decreased the risk of severe mental health issues due to sedentary behavior at work: −8% (−13 to −2%) for fully adjusted odds ratio and −8% (−13 to −3%) for crude odds ratio, per 10%-high-education (Fig 5). Those results were not significant in all other models. Sex, BMI, and marital status did not influence our results.

## Discussion

The main findings were that occupational sedentary behavior was associated with an increased the risk of mental health issues by 34% to 85% depending on models used. Mental health issues were psychological distress, depression, anxiety, stress, deep sadness, and burnout. Despite insufficient data precluding further analysis, work environment may be a main influencing factor for mental health issues when exposed to sedentary behavior at work. Inconsistent results precluded robust conclusion for variables that may further influence this risk, such as age and education.

### Occupational sedentary behavior and mental health risk

Our main finding was that occupational sedentary behavior was associated with an increased risk of mental health, consistently in all models. Study design did not significantly influence the results. All cross-sectional studies included in our meta-analysis showed an association of prolonged sitting time at work with mental health. Furthermore, two out of three longitudinal studies included showed an association of prolonged sitting time at work with mental health [20,34]. Result remains consistent even after adjusting with the level of physical activity, job stressors, and other covariates [49]. However, other studies reported contradictory results. Hagger-Johnson found that mental disorder symptoms were driven by sitting time at work in a cross sectional, but not in longitudinal analysis [5]. They found prior symptoms of mental disorder were linked to longer sitting in leisure time. Other study also did not find the association between sedentary time and depressive symptoms, anxiety or cognitive performance after a follow-up period of 5.7 years [56]. Additionally, a 13-year cohort study demonstrated differential effects between passive (e.g., watching TV) and active (e.g., reading, office work) sedentary behaviors on major depressive disorder indicating that mentally active sedentary behaviors were protective factor. Interestingly, these effects are largely independent of physical activity levels [57]. A possible explanation may be that mentally active tasks, such as working and sitting in a meeting that happened in the working environment is usually linked to self-reliance, achievement, and sense of autonomy, as well as active participation and engagement in working environment [58]. On the other side, mentally passive sedentary behavior, such as TV-viewing was associated with minimal cognitive effort [57]. Similar to the “physical activity paradox” the fact that leisure time physical activity is beneficial for health while occupational physical activity can be detrimental [59], further studies should also compare the effect of sedentary time at work with the effects of leisure sedentary time on mental health. Sedentary time at work might be less detrimental than leisure sedentary time because it is related to mentally active tasks – while leisure sedentary time is passive tasks (watching TV, etc.). We can therefore have a sedentary paradox (occupational sedentary time less bad) opposite to the physical activity paradox (occupational physical activity worst). In the literature, no studies concomitantly assessed all types of mental health issues that could be linked with occupational sedentary behavior, but they rather focused on a dedicated pathology. Our meta-analyses included psychological distress, depression, anxiety, stress, deep sadness, and burnout, giving a global overview, but lack of comparisons between pathologies. Further studies should deeply explore the effect the of occupational sedentary behavior on each type of mental health.

### Occupational factors and mental health risk

Mental health issues associated with occupation have been well-defined in many studies. In our meta-analysis, we identified various types of occupations, including police officers, healthcare workers, caregivers, corporate employees, and civil servants. However, insufficient data precluded comparisons between occupational groups. Nevertheless, a study found that white-collar workers who spent more than 3 h/day had a higher risk of high perceived stress, while no impact was found on blue-collar workers [25]. This result was consistent with a study included in our review revealing the association between long sedentary behavior and poor mental health in white-collar workers, but this association was not observed in blue-collar workers [51]. It can be explained by the fact that white-collar workers usually have higher psychological demands due to their high position that correlated with self-perceived work stress [60]. Besides, prolonged sitting time was also associated with lower work engagement among white collar-workers [51]. This finding may also be due to lower sedentary levels in blue-collars compared to white-collars, that eventually did not affect their mental health [51]. Interestingly, we also included one study focusing on remote workers [21]. A recent study in Japan published that individuals who worked in office spent higher energy expenditure and higher time sitting compared to those who work from home [61]. A randomized controlled trial aiming to intervene physical and mental well-being of the remote workers in India found that 95% of our participants did not meet the PA recommendation and 60% of them experienced mild to moderate anxiety, stress, and depression [21]. The intervention was only able to have low to moderate effect in their PA and sedentary level as well as a slight increase on stress, anxiety, and depression score. Thus, the effect of working at home on mental health are disputable as demonstrated in a systematic review, with some positive effect on stress due to family and work integration and less commuting, but also some negative effect on stress because of unclear work-home boundaries, social isolation, extended working hours, poor organizational support, as well as conflicts of work-family [62]. Seniority was also a protective factor against anxiety for workers as shown in a study [48]. Shift workers have a higher risk of depression and anxiety, that could be mediated by modifiable factors such as sedentary time, smoking, sleep duration, and BMI [55].

### Sociodemographics and mental health risk

Despite inconsistent results in our meta-analysis, age may increase the risk of mental health issue for workers with high sedentary behavior at work. In a study included in our meta-analysis, older police officers had a twice greater risk of deep sadness possibly due to the cumulative effect of all stressors, violence, and psychological traumatisms experienced during their careers [48]. In contrary, a study reported that younger age was associated with greater psychological distress alongside with higher occupational sedentary and less leisure time physical activity [50]. The fact that detrimental effect of sedentary behavior on mental health may vary depending on age should be further studied. In occupational context, the relation between age and increased risk of mental health issues could be explained by a sense of job insecurity, job strain, and low job satisfaction, although occupational sedentary behavior was not assessed [63]. Despite the absence of effect in our study, sex could also be an influencing factor on the risk of mental health due to occupational sedentary behavior. Some studies showed that women had higher levels of stress, anxiety, and depression compared to men [20,21], possibly linked with shared responsibility inequities in parenting and household tasks [64]. A study in police officers also showed sex-specific response to traumatic situations and stressful events with higher self-blame and denials in women [48,65]. Additionally, our result showed that higher education was a protective factor reducing the risk of mental health when exposed to sedentary behavior at work. A study assessing sitting time in older office workers showed that lower education levels were significantly related to higher perceived stress and more depressive symptoms [63] suggesting that psychological health is improved by education [66]. In the literature, while lower education was linked with prolonged sedentary behavior [67,68], workers with higher education were more likely to perceive their work as more stressful [25]. Yet no effect found in our study, higher BMI was associated with depression and anxiety through several psychological processes such as weight discrimination [69,70], body dissatisfaction [71], emotional eating, poor emotion regulation, and low exercise confidence [72]. Although no effects were found in our study, the relation between stress and marital status was always conflicting, where married individuals or couples being associated with lower [22,23] or greater psychological distress [50]. A modulator of this relation could be sex, marriage being a protective health factor mostly for men [73,74]. Even if insufficient data precluded to search for an influence of physical activity on mental health, low levels of physical activity were frequently associated in the literature with poor mental health [5,12,50] and lower productivity [21]. Interestingly, a meta-analysis found that physical activity at work was positively associated with mental health as well as mental ill-health [75]. Physical activity intervention generally improve quality of life, mood, and functional capacity [76]. In total, there are numerous articles that assessed the influence of sociodemographic on mental health or on sedentary behavior (in leisure time mostly and some at work), but there is a need for studies assessing the influence of sociodemographic on the impact of sedentary behavior on mental health, with a particular attention for distinguishing leisure time and occupational sedentary behavior, that may yield to different effect similar to the physical activity paradox [59].

### Strength and limitations

To the best of our knowledge, this is the first meta-analysis to quantitatively examine the association between occupational sedentary behavior and several mental health issues. We used a statistical method by computing four meta-analyses with the inclusion of only one risk per study (crude/less adjusted or fully/most adjusted, and pessimistic, i.e., the lowest risk or optimistic model, i.e., the highest risk) to reduce a bias of data selection. However, this study has several limitations. The number of included studies was small; however, we included a total sample size of more than 40,000 workers. Our meta-analysis inherits the bias from each included study, particularly bias of exposure and bias of outcome assessment. Studies included sitting time and mental health issues used mainly self-reported questionnaires, which may limit the quality of data. On the other hand, it also makes the evaluation reproducible. Most studies used self-questionnaire without supervision leading to bias such as incomplete information, non-disclosure or skipping questions due to social desirability bias or lack of self-awareness [77], that is frequently found in private or sensitive questions such as mental health condition [78]. The questionnaire used also varied between studies, making comparisons between studies more difficult. Additionally, most included studies were cross-sectional precluding to establish a cause-effect relation. Lack of longitudinal data did not permit to study long-term implications of sedentary behavior in the follow-up of mental health. Our population may seem heterogeneous but it is also in favor of the generalizability of our results. Moreover, we cover four continents except Africa, as it is unfortunately often the case in research [79]. Despite included studies used various cut-off both for sedentary behavior, we computed several sensitivity analyses using different cut-off. We conducted sensitivity analyses that yielded consistent results across various study designs. In our comprehensive meta-analysis model, some studies appeared multiple times with different data for the same outcome based on different adjustment models. Consequently, due to the lack of a clear consensus and despite the practice of including more effect sizes within each meta-analysis than the number of constituent studies, as seen in several articles published in high-ranked journals [80,81], the weighting of studies might have required adjustment [82]. This necessity supports the use of optimistic and pessimistic models, where each included article is represented by only one set of data. Further study can also involve device-based measures of sedentary behavior to determine the sitting time more accurately [83] and adjustments with physical activity and sleep levels. Recent studies have shown that physical activity, sedentary behavior and sleep are co-dependent, moreover in case of poor mental health, it should be considered together [84]. Despite these limitations, our meta-analysis is noteworthy to understand the effect of occupational sedentary behavior that may increase the risk mental health indicating the urgent need for mitigation at work.

## Conclusion

Our result demonstrated that occupational sedentary behavior was associated with an increased risk of mental health issues issues (psychological distress, depression, anxiety, stress, deep sadness, and burnout). The overall risk increased by 34% to 85% depending on models used. Inconsistent results precluded robust conclusion for variables that may further influence this risk. Considering the putative burden and health consequences linked to mental health, our study should encourage employers and employees to reduce sitting time at work and to build effective preventive strategy for well-being at work.

## Supporting information

S1 File Details of search strategy. (DOCX)

S1 Table PRISMA 2020 Checklist and PRISMA 2020 for Abstracts Checklist. (DOCX)

S2 File Details for evaluation of quality of included studies (using the SIGN and NOS), and associated checklists. (DOCX)

S2 Table Grading of Recommendation, Assessment, Development, and Evaluation (GRADE) instrument – Certainty of the evidence for our main outcome. HR: hazard ratio, MSA: muscle-strengthening activities, MVPA: moderate to vigorous physical activity, OR: odds ratio, SE: standard error.
(DOCX)

S1 Fig Detailed meta-analyses on the risk of mental health issues due to sedentary behavior. Blue lozenges represent the overall risk for each type of mental health issues after exposure to sedentary behavior. Dots represent the risk of mental health issues for each included study. The length of each horizontal line around the dots/lozenges represents their 95% confidence interval (95 CI). The black solid vertical line represents the null risk of mental health (with a value of 1). When horizontal lines cross the null vertical line, the risk of mental health is not significant. I-squared (%): percentage of heterogeneity between studies for each meta-analysis; Weight (%): Weight of each study for each meta-analysis.
(DOCX)

S2 Fig Meta-funnels for the risk of mental health issues due to occupational sedentary behavior, depending on groups: 1) risk of mental health (both intermediate and severe), 2) risk of severe mental health (all risks), 3) risk of severe mental health pessimistic model (fully adjusted risks), 4) risk of severe mental health pessimistic model (crude or less adjusted risks), 5) risk of severe mental health optimistic model (fully adjusted risks) and 6) risk of severe mental health optimistic model (crude or less adjusted risks). Each dot represents a single study, with its corresponding effect size (x axis) and its associated standard error of the effect estimate (y-axis). Large high-powered studies are placed towards the top, and smaller low-powered studies towards the bottom. The plot should ideally resemble a pyramid or inverted funnel, with scatter due to sampling variation. Studies outside funnel plot are likely to present bias.
(DOCX)

S1 Dataset Systematic review and meta-analysis of the impact of occupational sedentary behavior on mental health issues. (XLSX)
