# Supplementary material for: Impact of occupational sedentary behavior on mental health: A systematic review and meta-analysis
Source: PLoS One. 2025 Aug 20;20(8):e0328678. doi: 10.1371/journal.pone.0328678 (PMC12367128; doi:10.1371/journal.pone.0328678)
Supplement: S1 File — (DOCX) [file pone.0328678.s001.docx]

**Supporting Information**

# S1 File. Details of search strategy

Date of search: August 2024

Filter Language = none

Filter Dates = none

Total number of results (hits) across all databases: 5359

Number of results (hits) once duplicates removed: 241

| **Database** | **Results** | **Duplicate** |
| --- | --- | --- |
| Pubmed | 2745 | 129 |
| Embase | 691 | 35 |
| Cohcrane | 848 | 44 |
| Psycinfo | 1075 | 54 |
| **Total** | **5359** | **241** |

**PubMed**

| **#** | **Keywords** | **Results** |
| --- | --- | --- |
| 1 | (("Sedentary"[MeSH Terms] OR "Sedentary" [Text Word] OR "Sedentarity" [Text Word] OR "Sitting"[Text Word] OR "Sedentariness" [Text Word] OR "inactive lifestyle"[Text Word] OR "lying down"[Text Word] OR "Sedentary lifestyle"[Text Word] OR "Sedentary behaviour"[Text Word] OR "Sedentary behaviours"[Text Word] OR "Sedentary behaviour"[Text Word] OR "Sedentary behaviours"[Text Word] OR "Sedentary, work"[Text Word] OR "Sedentarity at work"[Text Word] OR "Occupational sedentary behaviour"[Text Word] OR "Occupational sedentary behaviour"[Text Word] OR "Screen time"[Text Word] OR "Screen"[Text Word])) | 256 251 |
| 2 | (("worker"[MeSH Terms] OR "worker"[Text Word] OR "workers"[Text Word] OR "work"[Text Word] OR "works"[Text Word] OR "job" [Text Word] OR "jobs" [Text Word] OR "office"[Text Word] OR "offices"[Text Word] OR "occupation"[Text Word] OR "occupations"[Text Word] OR "officers"[Title/Abstract:~3] OR officers"[Title/Abstract:~3])) | 1 951 439 |
| 3 | (("depression"[MeSH Terms] OR "depression risk"[MeSH Terms] OR "depression risks"[MeSH Terms] OR "depression"[Text Word] OR "depression risk"[Text Word] OR "depression risks"[Text Word] OR "symptoms of depression"[Text Word] OR "stress"[Text Word] OR "anxiety" [Text Word] OR "anxious"[Text Word] OR "mental health "[Text Word] OR "stress" [Text Word])) | 2 052 640 |
| **4** | **1 and 2 and 3** | 2745 |

**Embase**

| **#** | **Keywords** | **Results** |
| --- | --- | --- |
| 1 | ('sedentary lifestyle'/exp OR 'sedentary lifestyle' OR 'sedentary behavior':ti,ab,kw OR 'disuse':ti,ab,kw OR 'sedentary lifestyles':ti,ab,kw OR 'sedentary life style':ti,ab,kw OR 'sedentary behaviors':ti,ab,kw OR 'behavior sedentary':ti,ab,kw OR 'lifestyles sedentary':ti,ab,kw OR 'sedentary lifestyle':ti,ab,kw OR 'inactivity physical':ti,ab,kw OR 'lifestyle sedentary':ti,ab,kw OR 'lack of physical exercise':ti,ab,kw OR 'sedentariness':ti,ab,kw OR 'lack of physical activity':ti,ab,kw OR 'sedentary':ti,ab,kw OR 'sitting':ti,ab,kw) | Number of hits per term is not available as the search in Embase was made by the library of “Université Paris Cité”. We are thankful to “Université Paris Cité” for their help in this study. |
| 2 | ('stress near/3 occupational':ti,ab,kw OR 'stress near/3 workers':ti,ab,kw OR 'stress near/3 work':ti,ab,kw OR 'stress near/3 workplace':ti,ab,kw OR (('occupational disease'/exp OR 'occupational disease' OR 'stress related diseases':ti,ab,kw OR 'occupational illnesses':ti,ab,kw OR 'disorders occupational':ti,ab,kw OR 'occupational diseases':ti,ab,kw OR 'diseases occupational':ti,ab,kw OR 'illnesses occupational':ti,ab,kw OR 'disease occupational':ti,ab,kw OR 'occupational health':ti,ab,kw OR 'industrial diseases':ti,ab,kw OR 'occupational disease':ti,ab,kw OR 'industrial medicine':ti,ab,kw OR 'occupational disorder':ti,ab,kw OR 'occupational health'/exp OR 'occupational health' OR 'occupational injuries':ti,ab,kw OR 'occupational safety':ti,ab,kw OR 'employee health':ti,ab,kw OR 'health occupational':ti,ab,kw OR 'hygiene industrial':ti,ab,kw OR 'health industrial':ti,ab,kw OR 'health employee':ti,ab,kw OR 'industrial hygiene':ti,ab,kw OR 'safety occupational':ti,ab,kw OR 'industrial health':ti,ab,kw OR 'named groups by occupation'/exp OR 'named groups by occupation' OR 'personnel':ti,ab,kw OR 'occupational groups':ti,ab,kw OR 'employee':ti,ab,kw OR 'workers':ti,ab,kw OR 'occupational cohort':ti,ab,kw OR 'occupational group':ti,ab,kw OR 'groups occupational':ti,ab,kw OR 'employees':ti,ab,kw OR 'group occupational':ti,ab,kw OR 'worker':ti,ab,kw OR 'working':ti,ab,kw OR 'professionals':ti,ab,kw OR 'professional':ti,ab,kw OR 'workplace'/exp OR 'workplace' OR 'work location':ti,ab,kw OR 'place work':ti,ab,kw OR 'work-sites':ti,ab,kw OR 'workplaces':ti,ab,kw OR 'place of work':ti,ab,kw OR 'work places':ti,ab,kw OR 'job sites':ti,ab,kw OR 'locations work':ti,ab,kw OR 'location work':ti,ab,kw OR 'workplace':ti,ab,kw OR 'work place':ti,ab,kw OR 'work-site':ti,ab,kw OR 'job site':ti,ab,kw OR 'work locations':ti,ab,kw OR 'places work':ti,ab,kw OR 'worksites':ti,ab,kw OR 'worksite':ti,ab,kw OR 'site job':ti,ab,kw OR 'work'/exp OR 'work' OR 'labor':ti,ab,kw OR 'work':ti,ab,kw OR 'occupational':ti,ab,kw) |  |
| 3 | ('depression'/exp OR 'depression' OR 'depression mental':ti,ab,kw OR 'depression nos':ti,ab,kw OR 'syndromes depressive':ti,ab,kw OR 'feeling sad':ti,ab,kw OR 'depressed':ti,ab,kw OR 'state depressive':ti,ab,kw OR 'dysthymic disorder':ti,ab,kw OR 'dysthymia'/exp OR 'dysthymia' OR 'mood depression':ti,ab,kw OR 'depression disorder':ti,ab,kw OR 'depressive disorder':ti,ab,kw OR 'decreased mood':ti,ab,kw OR 'depressed mood':ti,ab,kw OR 'feeling down':ti,ab,kw OR 'disorder;depressive':ti,ab,kw OR 'depression disease':ti,ab,kw OR 'depressions':ti,ab,kw OR 'depressive syndromes':ti,ab,kw OR 'central depression':ti,ab,kw OR 'disorder;depression':ti,ab,kw OR 'feeling depressed':ti,ab,kw OR 'depressive symptoms':ti,ab,kw OR 'disorders depressive':ti,ab,kw OR 'unipolar depression':ti,ab,kw OR 'major depression'/exp OR 'major depression' OR 'depressive state':ti,ab,kw OR 'emotional depression':ti,ab,kw OR 'depressivity':ti,ab,kw OR 'symptoms depressive':ti,ab,kw OR 'feeling blue':ti,ab,kw OR 'syndrome depressive':ti,ab,kw OR 'mental depression':ti,ab,kw OR 'mood depressions':ti,ab,kw OR 'low mood':ti,ab,kw OR 'depression general':ti,ab,kw OR 'depressive symptom':ti,ab,kw OR 'depressive disease':ti,ab,kw OR 'depressive illness':ti,ab,kw OR 'depressive syndrome':ti,ab,kw OR 'clinical depression':ti,ab,kw OR 'depression':ti,ab,kw OR 'symptom depressive':ti,ab,kw OR 'major depressive disorder':ti,ab,kw OR 'depressed state':ti,ab,kw OR 'mental health'/exp OR 'mental health' OR 'healthmental':ti,ab,kw OR 'mental hygiene':ti,ab,kw OR 'hygiene mental':ti,ab,kw OR 'emotional health':ti,ab,kw OR 'emotional stability'/exp OR 'emotional stability' OR 'mental health':ti,ab,kw OR 'mental well-being':ti,ab,kw OR 'psychological well-being'/exp OR 'psychological well-being' OR 'anxiety'/exp OR 'anxiety' OR 'anxiety':ti,ab,kw OR 'anxious':ti,ab,kw OR 'anxieties':ti,ab,kw OR 'work-related stress':ti,ab,kw OR 'job-related stress':ti,ab,kw OR 'stresses job':ti,ab,kw OR 'workplace stresses':ti,ab,kw OR 'stress job':ti,ab,kw OR 'job-related stresses':ti,ab,kw OR 'job stress'/exp OR 'job stress' OR 'professional stress':ti,ab,kw OR 'workplace stress':ti,ab,kw OR 'stress occupational':ti,ab,kw OR 'work place stresses':ti,ab,kw OR 'work-related stresses':ti,ab,kw OR 'bullying workplace':ti,ab,kw OR 'job stresses':ti,ab,kw OR 'job stress':ti,ab,kw OR 'work place stress':ti,ab,kw OR 'stress workplace':ti,ab,kw OR 'occupational stresses':ti,ab,kw OR 'stresses professional':ti,ab,kw OR 'stress work related':ti,ab,kw OR 'occupational stress':ti,ab,kw OR 'professional stresses':ti,ab,kw OR 'stress job related':ti,ab,kw OR 'stress professional':ti,ab,kw) |  |
| 4 | ([article]/lim OR [article in press]/lim) |  |
| **5** | **1 and 2 and 3 AND 4** | **691** |

**Cochrane**

| **#** | **Keywords** | **Results** |
| --- | --- | --- |
| 1 | ("occupational" OR "workers") | 113847 |
| 2 | ("sedentary behaviour") | 28179 |
| 3 | ("mental health" OR depression OR stress OR anxiety) | 244905 |
| **4** | **1 and 2 and 3** | **848** |

**Psycinfo**

| **#** | **Keywords** | **Results** |
| --- | --- | --- |
| 1 | ("occupational" OR "workers") | 273 390 |
| 2 | ("sedentary behaviour") | 2 722 |
| 3 | ("mental health" OR depression OR stress OR anxiety) | 1 455 084 |
| **4** | **1 and 2 and 3** | **1 075** |
