# Supplementary material for: Impact of occupational sedentary behavior on mental health: A systematic review and meta-analysis
Source: PLoS One. 2025 Aug 20;20(8):e0328678. doi: 10.1371/journal.pone.0328678 (PMC12367128; doi:10.1371/journal.pone.0328678)
Supplement: S2 File — (DOCX) [file pone.0328678.s003.docx]

**Supporting Information**

# S3 File. Details for evaluation of quality of included studies (using the SIGN and NOS), and associated checklists.

Details of quality of included studies using the SIGN

Summary of quality of included studies

using the NOS checklist

Risk of bias using

The New-Ottawa Scale (NOS) checklist

We used The New-Ottawa Scale (NOS) for cohort study that consists of three dimension – selection, comparability, and outcome – for a total of nine items scored one point each and providing a maximum score of 9.

Summary bias

(cohort studies n =7)


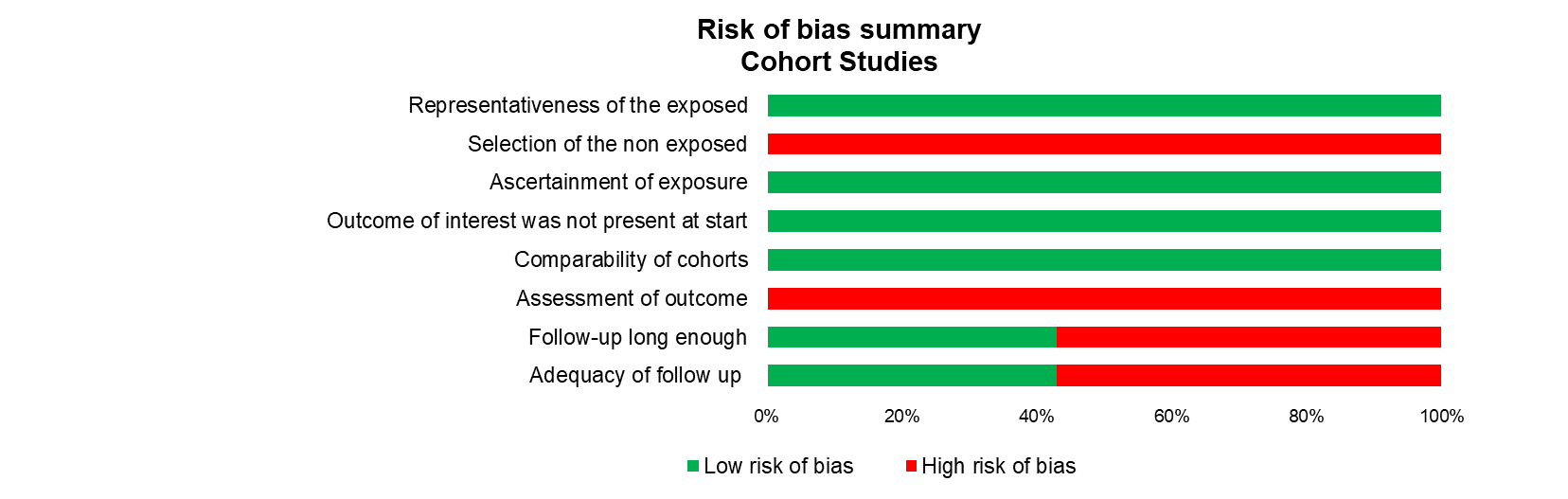


Details of quality of included studies

| 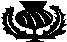 S I G N | | Methodology Checklist 3: Cohort studies | | | |
| --- | --- | --- | --- | --- | --- |
| Study identification (*Include author, title, year of publication, journal title, pages*) | | | | | |
| Guideline topic: | | | Key Question No: | | Reviewer: |
| Before completing this checklist, consider:  Is the paper really a cohort study? If in doubt, check the study design algorithm available from SIGN and make sure you have the correct checklist.  Is the paper relevant to key question? Analyse using PICO (Patient or Population Intervention Comparison Outcome). IF NO REJECT (give reason below). IF YES complete the checklist.. | | | | | |
| Reason for rejection: 1. Paper not relevant to key question □ 2. Other reason □ (please specify):  Please note that a retrospective study (ie a database or chart study) cannot be rated higher than +. | | | | | |
| Section 1: Internal validity | | | | | |
| *In a well conducted cohort study:* | | | | Does this study do it? | |
| 1.1 | The study addresses an appropriate and clearly focused question.^i^ | | | Yes □  Can’t say □ | No □ |
| Selection of subjects | | | | | |
| 1.2 | The two groups being studied are selected from source populations that are comparable in all respects other than the factor under investigation.^ii^ | | | Yes □  Can’t say □ | No □  Does not apply □ |
| 1.3 | The study indicates how many of the people asked to take part did so, in each of the groups being studied.^iii^ | | | Yes □ | No □  Does not apply □ |
| 1.4 | The likelihood that some eligible subjects might have the outcome at the time of enrolment is assessed and taken into account in the analysis.^iv^ | | | Yes □  Can’t say □ | No □  Does not apply □ |
| 1.5 | What percentage of individuals or clusters recruited into each arm of the study dropped out before the study was completed.^v^ | | |  | |
| 1.6 | Comparison is made between full participants and those lost to follow up, by exposure status.^vi^ | | | Yes □  Can’t say □ | No □  Does not apply □ |

**The Scottish Intercollegiate Guidelines Network (SIGN) checklist**

| ASSESSMENT | | | | |
| --- | --- | --- | --- | --- |
| 1.7 | The outcomes are clearly defined.^vii^ | Yes □  Can’t say □ | No □ | |
| 1.8 | The assessment of outcome is made blind to exposure status. If the study is retrospective this may not be applicable.^viii^ | Yes □  Can’t say □ | No □  Does not apply □ | |
| 1.9 | Where blinding was not possible, there is some recognition that knowledge of exposure status could have influenced the assessment of outcome.^ix^ | Yes □  Can’t say □ | No □  □ | |
| 1.10 | The method of assessment of exposure is reliable.^x^ | Yes □  Can’t say □ | No □ | |
| 1.11 | Evidence from other sources is used to demonstrate that the method of outcome assessment is valid and reliable.^xi^ | Yes □  Can’t say □ | No □  Does not apply□ | |
| 1.12 | Exposure level or prognostic factor is assessed more than once.^xii^ | Yes □  Can’t say □ | No □  Does not apply □ | |
| CONFOUNDING | | | | |
| 1.13 | The main potential confounders are identified and taken into account in the design and analysis.^xiii^ | Yes □  Can’t say □ | No □ | |
| STATISTICAL ANALYSIS | | | | |
| 1.14 | Have confidence intervals been provided?^xiv^ | Yes □ | No □ | |
| Section 2: OVERALL ASSESSMENT OF THE STUDY | | | | |
| 2.1 | How well was the study done to minimise the risk of bias or confounding?^xv^ | High quality (++) □  Acceptable (+) □  Unacceptable – reject 0 | | |
| 2.2 | Taking into account clinical considerations, your evaluation of the methodology used, and the statistical power of the study, do you think there is clear evidence of an association between exposure and outcome? | Yes □  Can’t say □ | | No □ |
| 2.3 | Are the results of this study directly applicable to the patient group targeted in this guideline? | Yes □ | | No □ |
| 2.4 | Notes. Summarise the authors conclusions. Add any comments on your own assessment of the study, and the extent to which it answers your question and mention any areas of uncertainty raised above. | | | |

i Unless a clear and well defined question is specified in the report of the review, it will be difficult to assess how well it has met its objectives or how relevant it is to the question you are trying to answer on the basis of the conclusions.

ii This relates to selection bias.* It is important that the two groups selected for comparison are as similar as possible in all characteristics except for their exposure status, or the presence of specific prognostic factors or prognostic markers relevant to the study in question.

iii This relates to selection bias.* The participation rate is defined as the number of study participants divided by the number of eligible subjects, and should be calculated separately for each branch of the study. A large difference in participation rate between the two arms of the study indicates that a significant degree of selection bias* may be present, and the study results should be treated with considerable caution.

iv If some of the eligible subjects, particularly those in the unexposed group, already have the outcome at the start of the trial the final result will be subject to performance bias.* A well conducted study will attempt to estimate the likelihood of this occurring, and take it into account in the analysis through the use of sensitivity studies or other methods.

v This question relates to the risk of attrition bias.*The number of patients that drop out of a study should give concern if the number is very high. Conventionally, a 20% drop out rate is regarded as acceptable, but in observational studies conducted over a lengthy period of time a higher drop out rate is to be expected. A decision on whether to downgrade or reject a study because of a high drop out rate is a matter of judgement based on the reasons why people dropped out, and whether drop out rates were comparable in the exposed and unexposed groups. Reporting of efforts to follow up participants that dropped out may be regarded as an indicator of a well conducted study.

vi For valid study results, it is essential that the study participants are truly representative of the source population. It is always possible that participants who dropped out of the study will differ in some significant way from those who remained part of the study throughout. A well conducted study will attempt to identify any such differences between full and partial participants in both the exposed and unexposed groups. This relates to the risk of attrition bias.* Any unexplained differences should lead to the study results being treated with caution.

vii This relates to the risk of detection bias.* Once enrolled in the study, participants should be followed until specified end points or outcomes are reached. In a study of the effect of exercise on the death rates from heart disease in middle aged men, for example, participants might be followed up until death, or until reaching a predefined age. If outcomes and the criteria used for measuring them are not clearly defined, the study should be rejected.

viii This relates to the risk of detection bias.* If the assessor is blinded to which participants received the exposure, and which did not, the prospects of unbiased results are significantly increased. Studies in which this is done should be rated more highly than those where it is not done, or not done adequately.

ix This relates to the risk of detection bias.* Blinding is not possible in many cohort studies. In order to asses the extent of any bias that may be present, it may be helpful to compare process measures used on the participant groups - e.g. frequency of observations, who carried out the observations, the degree of detail and completeness of observations. If these process measures are comparable between the groups, the results may be regarded with more confidence.

x This relates to the risk of detection bias.* A well conducted study should indicate how the degree of exposure or presence of prognostic factors or markers was assessed. Whatever measures are used must be sufficient to establish clearly that participants have or have not received the exposure under investigation and the extent of such exposure, or that they do or do not possess a particular prognostic marker or factor. Clearly described, reliable measures should increase the confidence in the quality of the study

xi This relates to the risk of detection bias.* The primary outcome measures used should be clearly stated in the study. If the outcome measures are not stated, or the study bases its main conclusions on secondary outcomes, the study should be rejected. Where outcome measures require any degree of subjectivity, some evidence should be provided that the measures used are reliable and have been validated prior to their use in the study.

xii This relates to the risk of detection bias.* Confidence in data quality should be increased if exposure level is measured more than once in the course of the study. Independent assessment by more than one investigator is preferable.

xiii Confounding is the distortion of a link between exposure and outcome by another factor that is associated with both exposure and outcome. The possible presence of confounding factors is one of the principal reasons why observational studies are not more highly rated as a source of evidence. The report of the study should indicate which potential confounders have been considered, and how they have been assessed or allowed for in the analysis. Clinical judgement should be applied to consider whether all likely confounders have been considered. If the measures used to address confounding are considered inadequate, the study should be downgraded or rejected, depending on how serious the risk of confounding is considered to be. A study that does not address the possibility of confounding should be rejected.

xiv Confidence limits are the preferred method for indicating the precision of statistical results, and can be used to differentiate between an inconclusive study and a study that shows no effect. Studies that report a single value with no assessment of precision should be treated with extreme caution.

xv Rate the overall methodological quality of the study, using the following as a guide: High quality (++): Majority of criteria met. Little or no risk of bias. Results unlikely to be changed by further research. Acceptable (+): Most criteria met. Some flaws in the study with an associated risk of bias, Conclusions may change in the light of further studies. Low quality (0): Either most criteria not met, or significant flaws relating to key aspects of study design. Conclusions likely to change in the light of further studies.

The NOS checklist

for cohort studies

Note: A study can be awarded a maximum of one star for each numbered item within the Selection and Outcome categories. A maximum of two stars can be given for Comparability

Selection

1) Representativeness of the exposed cohort

a) truly representative of the average _______________ (describe) in the community 

b) somewhat representative of the average ______________ in the community 

c) selected group of users eg nurses, volunteers

d) no description of the derivation of the cohort

2) Selection of the non exposed cohort

a) drawn from the same community as the exposed cohort 

b) drawn from a different source

c) no description of the derivation of the non exposed cohort

3) Ascertainment of exposure

a) secure record (eg surgical records) 

b) structured interview 

c) written self report

d) no description

4) Demonstration that outcome of interest was not present at start of study

a) yes 

b) no

Comparability

1) Comparability of cohorts on the basis of the design or analysis

a) study controls for _____________ (select the most important factor) 

b) study controls for any additional factor  (This criteria could be modified to indicate specific control for a second important factor.)

Outcome

1) Assessment of outcome

a) independent blind assessment 

b) record linkage 

c) self report

d) no description

2) Was follow-up long enough for outcomes to occur

a) yes (select an adequate follow up period for outcome of interest) 

b) no

3) Adequacy of follow up of cohorts

a) complete follow up - all subjects accounted for 

b) subjects lost to follow up unlikely to introduce bias - small number lost - > ____ % (select an adequate %) follow up, or description provided of those lost) 

c) follow up rate < ____% (select an adequate %) and no description of those lost

d) no statement
