# Supplementary material for: Impact of occupational sedentary behavior on mental health: A systematic review and meta-analysis
Source: PLoS One. 2025 Aug 20;20(8):e0328678. doi: 10.1371/journal.pone.0328678 (PMC12367128; doi:10.1371/journal.pone.0328678)
Supplement: S2 Table — HR: hazard ratio, MSA: muscle-strengthening activities, MVPA: moderate to vigorous physical activity, OR: odds ratio, SE: standard error. (DOCX) [file pone.0328678.s004.docx]

**Supporting Information**

S4 Table. Grading of Recommendation, Assessment, Development, and Evaluation (GRADE) instrument – Certainty of the evidence for our main outcome

| Outcome | Study  design | n  studies (subgroup) | Certainty assessment | | | | Effect OR  95% CI | Quality of evidence^1^ | Importance of outcome |
| --- | --- | --- | --- | --- | --- | --- | --- | --- | --- |
|  |  |  | Risk of  bias | Risk of Inconsistency | Risk of Indirectness | Risk of imprecision |  |  |  |
| Risk of mental health issues (both intermediate and severe) | | | | | | | | | |
| Psychological distress | Cross-sectional,  prospective cohort | 2 (36) | high | moderate | high | moderate | 1.11 (1.00 to 1.21) | ⨁⨁⨁◯ | important |
| Depression | Cross-sectional,  retrospective cohort, prospective cohort | 3 (8) | low | low | low | moderate | 1.44 (1.15 to 1.72) | ⨁⨁◯◯ | important |
| Anxiety | Cross-sectional,  retrospective cohort | 2 (5) | moderate | low | low | moderate | 1.83 (1.20 to 2.46) | ⨁⨁◯◯ | important |
| Depression and anxiety | Cross-sectional | 1 (4) | high | low | low | moderate | 1.43 (1.31 to 1.54) | ◯◯◯◯ | important |
| Stress | Cross-sectional,  retrospective cohort | 2 (5) | low | low | low | moderate | 1.64 (1.20 to 2.08) | ⨁⨁◯◯ | important |
| Deep sadness | Cross-sectional | 1 (1) | significant | significant | high | significant | 1.85 (0.70 to 3.00) | ◯◯◯◯ | less important |
| Burnout | Cross-sectional | 1 (1) | significant | significant | high | significant | 2.49 (0.99 to 4.00) | ◯◯◯◯ | important |
| Overall |  | **7 (60)** | moderate | moderate | moderate | moderate | 1.34 (1.18 to 1.49) | ⨁⨁⨁◯ | important |
| Risk of severe mental health issues | | | | | | | | | |
| Psychological distress | Cross-sectional,  prospective cohort | 2 (14) | high | moderate | high | moderate | 0.94 (0.71 to 1.17) | ⨁⨁⨁◯ | important |
| Depression | Cross-sectional,  retrospective cohort, prospective cohort | 3 (7) | low | low | low | moderate | 1.33 (1.11 to 1.54) | ⨁⨁◯◯ | important |
| Anxiety | Cross-sectional,  retrospective cohort | 2 (4) | moderate | low | moderate | moderate | 1.84 (1.14 to 2.54) | ⨁◯◯◯ | important |
| Depression and anxiety | Cross-sectional | 1 (4) | high | low | low | moderate | 1.43 (1.31 to 1.54) | ◯◯◯◯ | important |
| Stress | Cross-sectional,  retrospective cohort | 2 (4) | low | low | low | moderate | 1.42 (1.16 to 1.68) | ⨁◯◯◯ | important |
| Deep sadness | Cross-sectional | 1 (1) | significant | significant | high | significant | 1.85 (0.70 to 3.00) | ◯◯◯◯ | less important |
| Burnout | Cross-sectional | 1 (1) | significant | significant | high | significant | 2.49 (0.99 to 4.00) | ◯◯◯◯ | important |
| Overall |  | **7 (35)** | moderate | moderate | moderate | moderate | 1.35 (1.12 to 1.58) | ⨁⨁⨁◯ | important |
| Fully adjusted odds ratio - Pessimistic model | | | | | | | | | |
| Psychological distress | Cross-sectional,  prospective cohort | 2 (3) | high | moderate | high | moderate | 1.39 (0.90 to 1.88) | ⨁◯◯◯ | important |
| Depression | Cross-sectional,  retrospective cohort, prospective cohort | 3 (3) | low | low | low | moderate | 1.11 (0.87 to 1.36) | ⨁⨁◯◯ | important |
| Anxiety | Cross-sectional,  retrospective cohort | 2 (2) | moderate | low | moderate | moderate | 1.38 (0.46 to 2.31) | ⨁◯◯◯ | important |
| Depression and anxiety | Cross-sectional | 1 (1) | high | low | low | moderate | 1.47 (1.23 to 1.71) | ◯◯◯◯ | important |
| Stress | Cross-sectional, retrospective cohort | 2 (2) | low | low | low | moderate | 1.66 (0.72 to 2.59) | ⨁◯◯◯ | important |
| Deep sadness | Cross-sectional | 1 (1) | significant | significant | high | significant | 1.85 (0.70 to 3.00) | ◯◯◯◯ | less important |
| Burnout | Cross-sectional | 1 (1) | significant | significant | high | significant | 2.49 (0.99 to 4.00) | ◯◯◯◯ | important |
| Overall |  | **7 (13)** | moderate | moderate | moderate | moderate | 1.39 (1.15 to 1.63) | ⨁⨁◯◯ | important |
| Crude or less adjusted odds ratio - Pessimistic model | | | | | | | | | |
| Psychological distress | Cross-sectional,  prospective cohort | 2 (3) | high | moderate | high | moderate | 1.37 (1.10 to 1.65) | ⨁◯◯◯ | important |
| Depression | Cross-sectional,  retrospective cohort, prospective cohort | 3 (3) | low | low | low | moderate | 1.11 (0.87 to 1.36) | ⨁⨁◯◯ | important |
| Anxiety | Cross-sectional,  retrospective cohort | 2 (2) | moderate | low | moderate | moderate | 1.38 (0.46 to 2.31) | ⨁◯◯◯ | important |
| Depression and anxiety | Cross-sectional | 1 (1) | moderate | low | low | moderate | 1.46 (1.24 to 1.68) | ◯◯◯◯ | important |
| Stress | Cross-sectional,  retrospective cohort | 2 (2) | low | low | low | moderate | 1.66 (0.72 to 2.59) | ⨁◯◯◯ | important |
| Deep sadness | Cross-sectional | 1 (1) | significant | significant | high | significant | 1.85 (0.70 to 3.00) | ◯◯◯◯ | less important |
| Burnout | Cross-sectional | 1 (1) | significant | significant | high | significant | 2.49 (0.99 to 4.00) | ◯◯◯◯ | important |
| Overall |  | **7 (13)** | moderate | moderate | moderate | moderate | 1.36 (1.13 to 1.59) | ⨁⨁◯◯ | important |
| Fully adjusted odds ratio - optimistic model | | | | | | | | | |
| Psychological distress | Cross-sectional,  prospective cohort | 2 (3) | high | moderate | high | moderate | 1.40 (0.90 to 1.88) | ⨁⨁◯◯ | important |
| Depression | Cross-sectional,  retrospective cohort, prospective cohort | 3 (3) | low | low | low | moderate | 1.56 (1.29 to 1.83) | ⨁◯◯◯ | important |
| Anxiety | Cross-sectional,  retrospective cohort | 2 (2) | moderate | low | moderate | moderate | 2.58 (1.64 to 3.53) | ⨁⨁◯◯ | important |
| Depression and anxiety | Cross-sectional | 1 (1) | high | low | low | moderate | 1.47 (1.23 to 1.71) | ◯◯◯◯ | important |
| Stress | Cross-sectional,  retrospective cohort | 2 (2) | low | low | low | moderate | 1.71 (1.04 to 2.38) | ◯◯◯◯ | important |
| Deep sadness | Cross-sectional | 1 (1) | significant | significant | high | significant | 1.85 (0.70 to 3.00) | ◯◯◯◯ | less important |
| Burnout | Cross-sectional | 1 (1) | significant | significant | high | significant | 2.49 (0.99 to 4.00) | ◯◯◯◯ | important |
| Overall |  | **7 (13)** | moderate | moderate | moderate | moderate | 1.85 (1.27 to 2.43) | ⨁⨁◯◯ | important |
| Crude or less adjusted odds ratio - optimistic model | | | | | | | | | |
| Psychological distress | Cross-sectional,  prospective cohort | 2 (3) | high | moderate | high | moderate | 1.37 (1.10 to 1.65) | ⨁⨁◯◯ | important |
| Depression | Cross-sectional,  retrospective cohort, prospective cohort | 3 (3) | low | low | low | moderate | 1.56 (1.29 to 1.83) | ⨁◯◯◯ | important |
| Anxiety | Cross-sectional,  retrospective cohort | 2 (2) | moderate | low | moderate | moderate | 2.58 (1.64 to 3.53) | ⨁⨁◯◯ | important |
| Depression and anxiety | Cross-sectional | 1 (1) | high | low | low | moderate | 1.46 (1.24 to 1.68) | ◯◯◯◯ | important |
| Stress | Cross-sectional,  retrospective cohort | 2 (2) | low | low | low | moderate | 1.71 (1.04 to 2.38) | ◯◯◯◯ | important |
| Deep sadness | Cross-sectional | 1 (1) | significant | significant | high | significant | 1.85 (0.70 to 3.00) | ◯◯◯◯ | less important |
| Burnout | Cross-sectional | 1 (1) | significant | significant | high | significant | 2.49 (0.99 to 4.00) | ◯◯◯◯ | important |
| Overall |  | **7 (11)** | moderate | moderate | moderate | moderate | 1.85 (1.26 to 2.43) | ⨁⨁◯◯ | important |
