# Supplementary material for: Impact of occupational sedentary behavior on mental health: A systematic review and meta-analysis
Source: PLoS One. 2025 Aug 20;20(8):e0328678. doi: 10.1371/journal.pone.0328678 (PMC12367128; doi:10.1371/journal.pone.0328678)

**Supporting Information**

# S5 Fig. Detailed of each meta-analysis on the risk of mental health issues due to occupational sedentary behaviour (only occupational in 4 studies and total i.e occupational and leisure in 3 studies) and (only the four studies that specifically assessed a risk associated with occupational sitting time).

*Blue lozenges represent the overall risk for each type of mental health issues after exposure to sedentary behaviour. Dots represent the risk of mental health issues for each included study. The length of each horizontal line around the dots/lozenges represents their 95% confidence interval (95CI). The black solid vertical line represents the null risk of mental health (with a value of 1). When horizontal lines cross the null vertical line, the risk of mental health is not significant.*

*I-squared (%): percentage of heterogeneity between studies for each meta-analysis; Weight (%): Weight of each study for each meta-analysis.*

Risk of mental health issues (both intermediate and severe)

**(**only occupational in 4 studies and total i.e occupational and leisure in 3 studies**)**


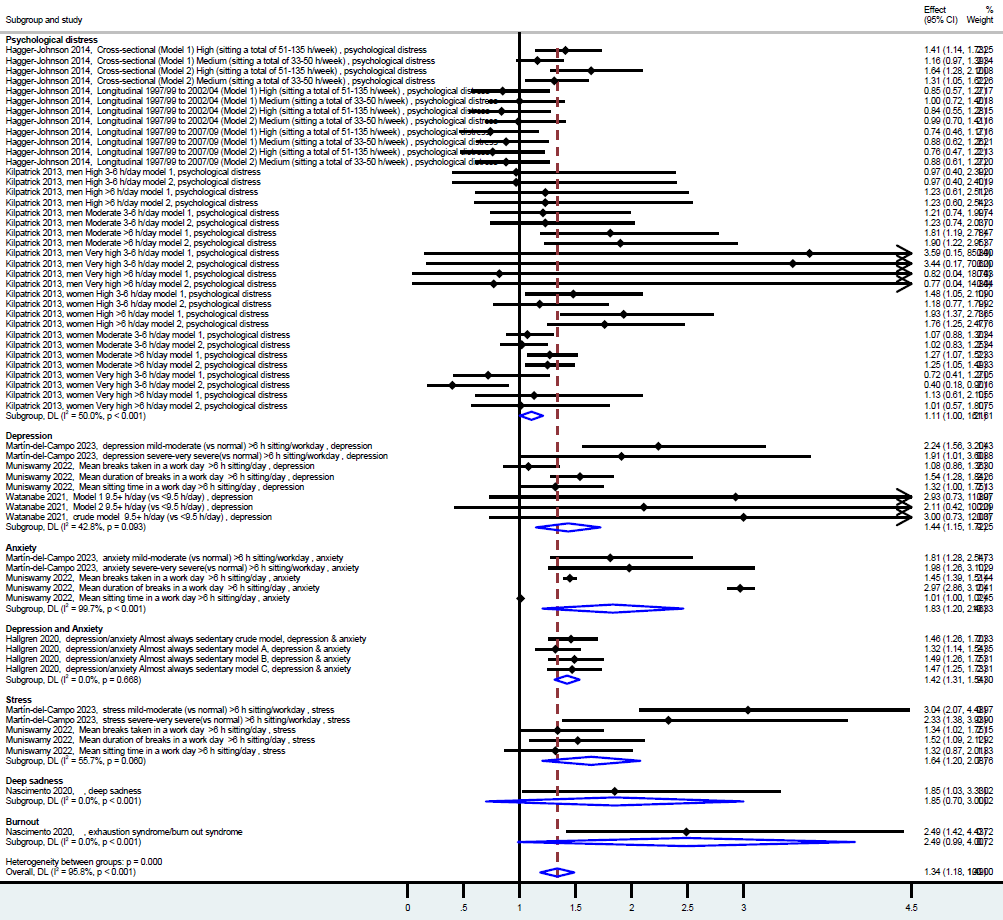


Risk of severe mental health issues

**(**only occupational in 4 studies and total i.e occupational and leisure in 3 studies**)**


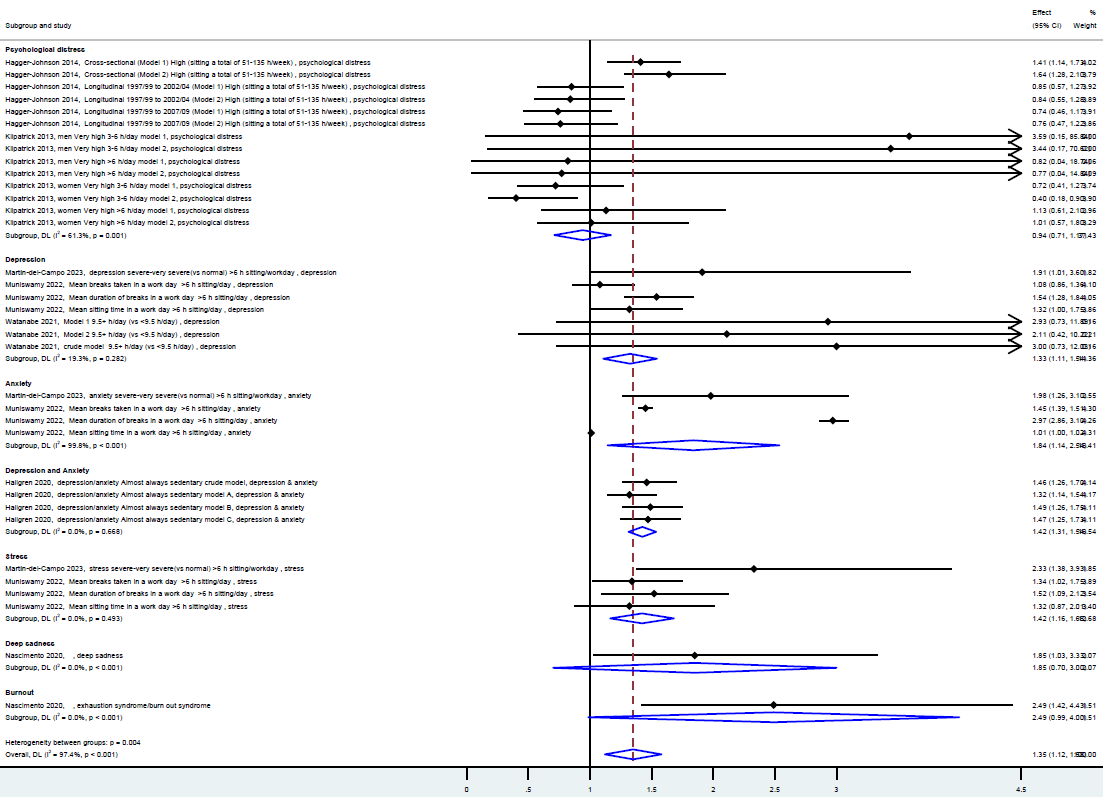


Risk of severe mental health issues

(Fully adjusted odds ratio - Pessimistic model)

**(**only occupational in 4 studies and total i.e occupational and leisure in 3 studies**)**


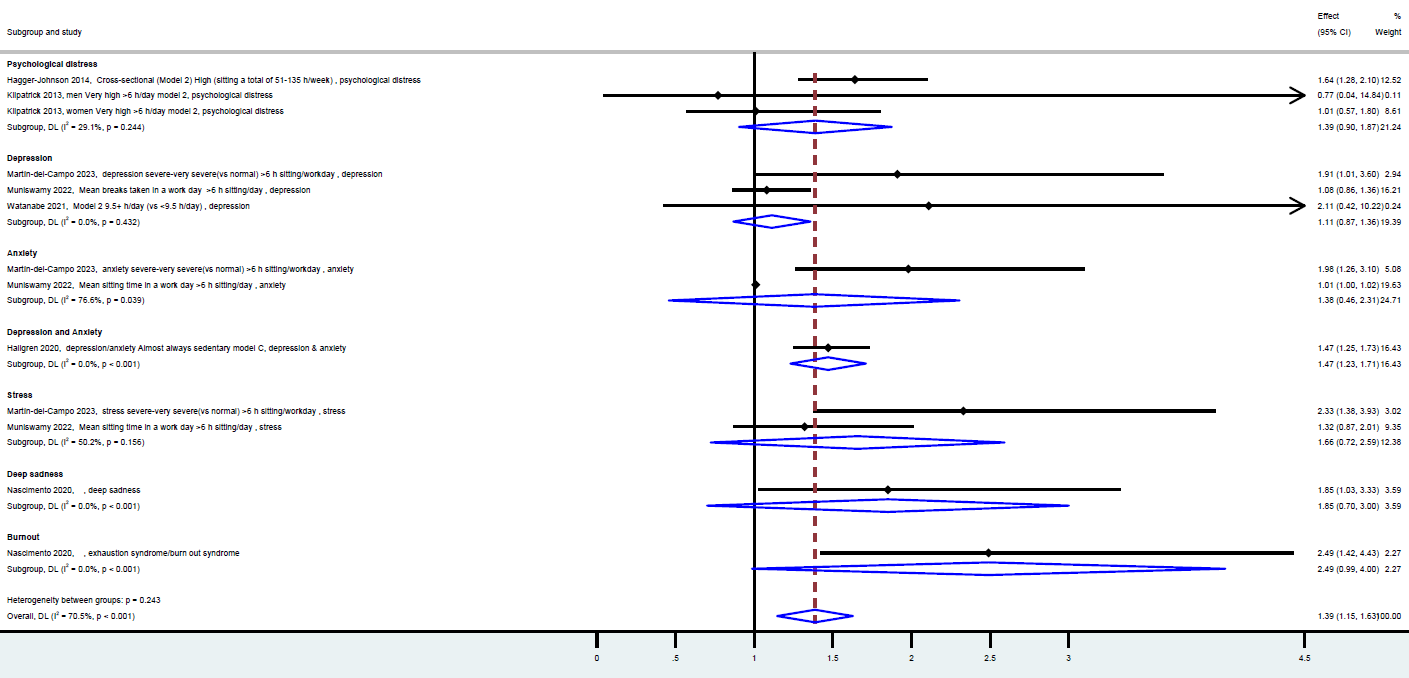


Risk of severe mental health issues

(Crude or less adjusted odds ratio - Pessimistic model)

**(**only occupational in 4 studies and total i.e occupational and leisure in 3 studies**)**


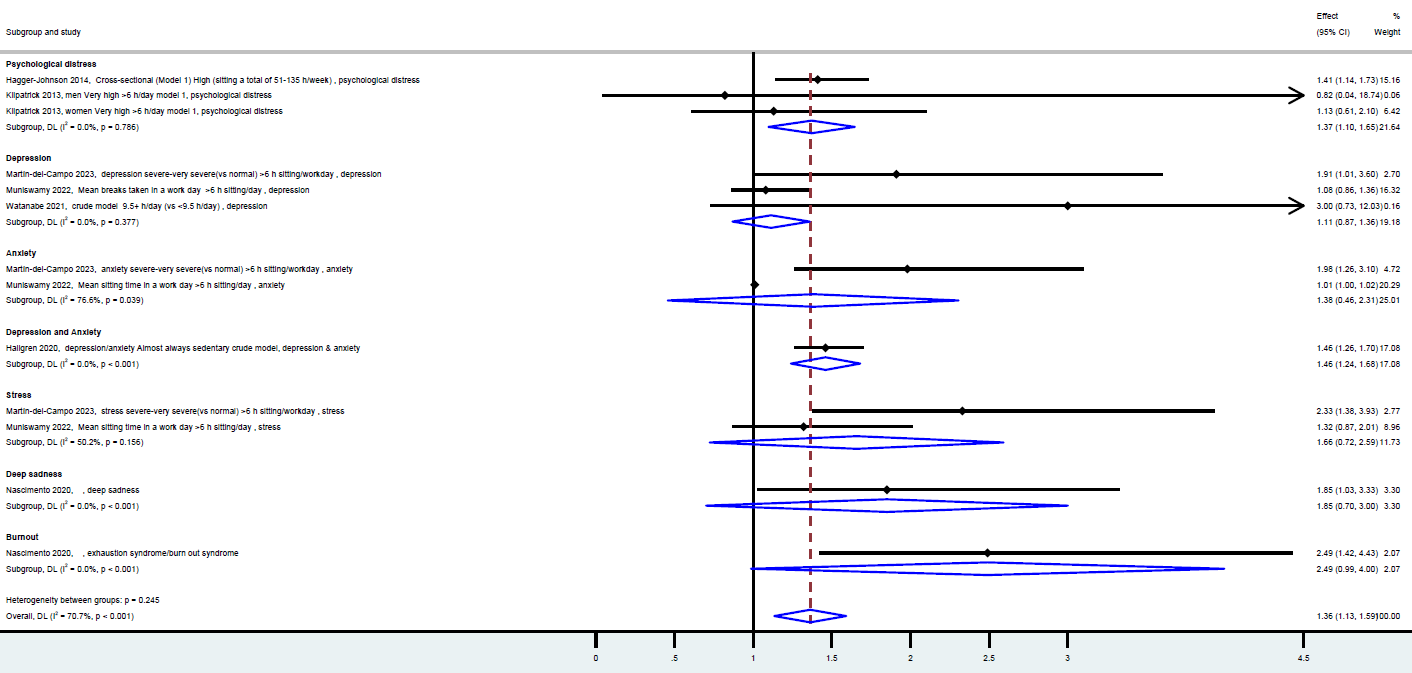


Risk of severe mental health issues

(Fully adjusted odds ratio – Optimistic model)

**(**only occupational in 4 studies and total i.e occupational and leisure in 3 studies**)**


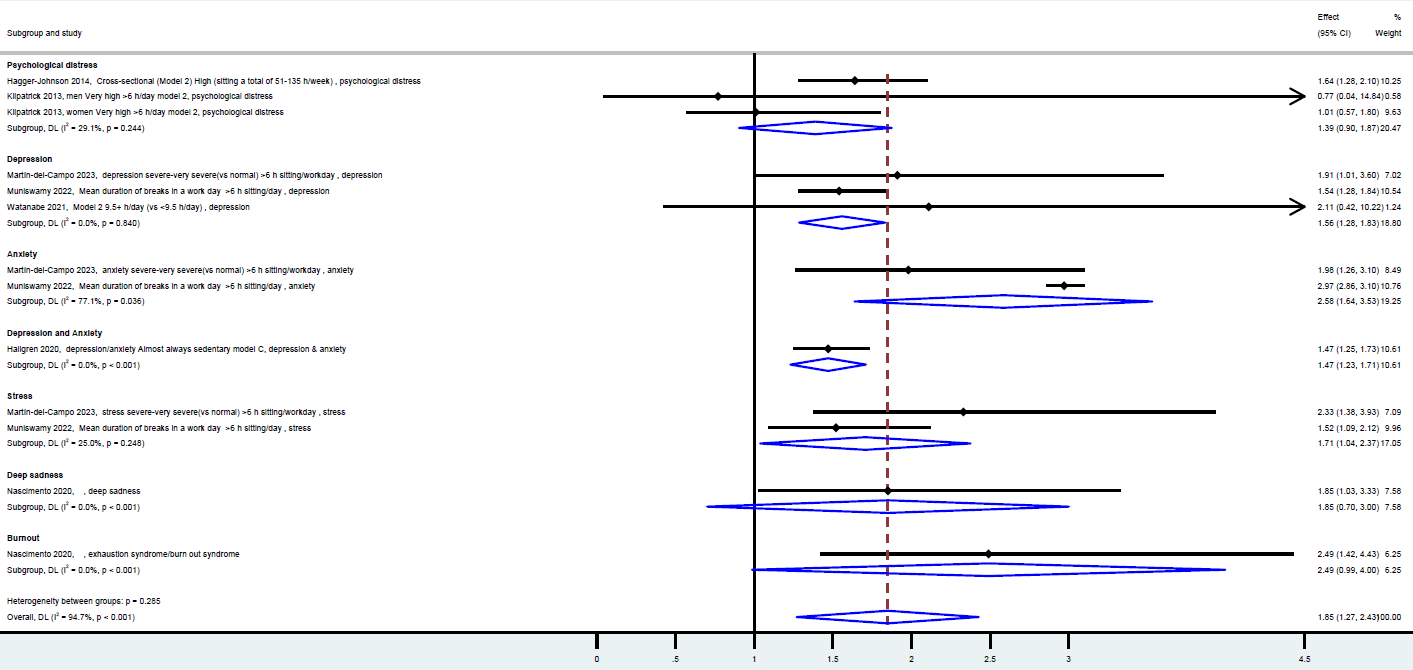


Risk of severe mental health issues

(Crude or less adjusted odds ratio - Optimistic model)

**(**only occupational in 4 studies and total i.e occupational and leisure in 3 studies**)**


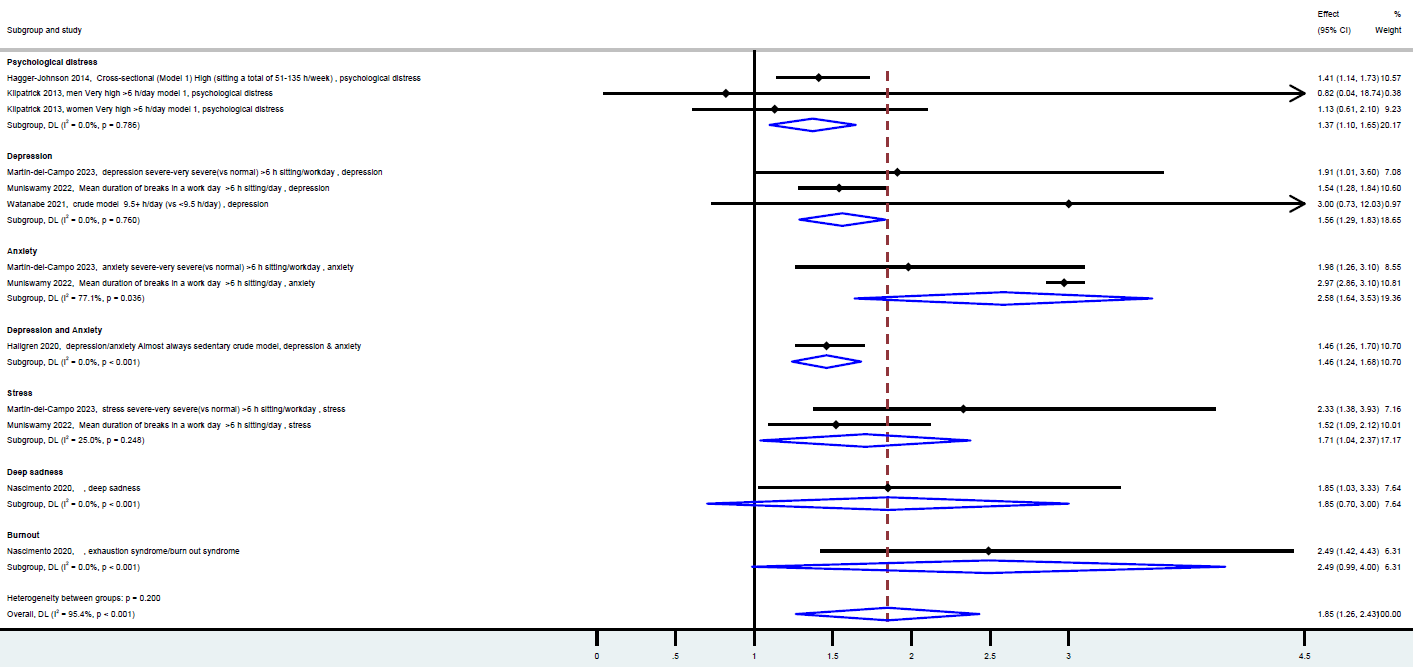


Risk of mental health issues (both intermediate and severe)

**(**only four studies assessing occupational sitting time**)**


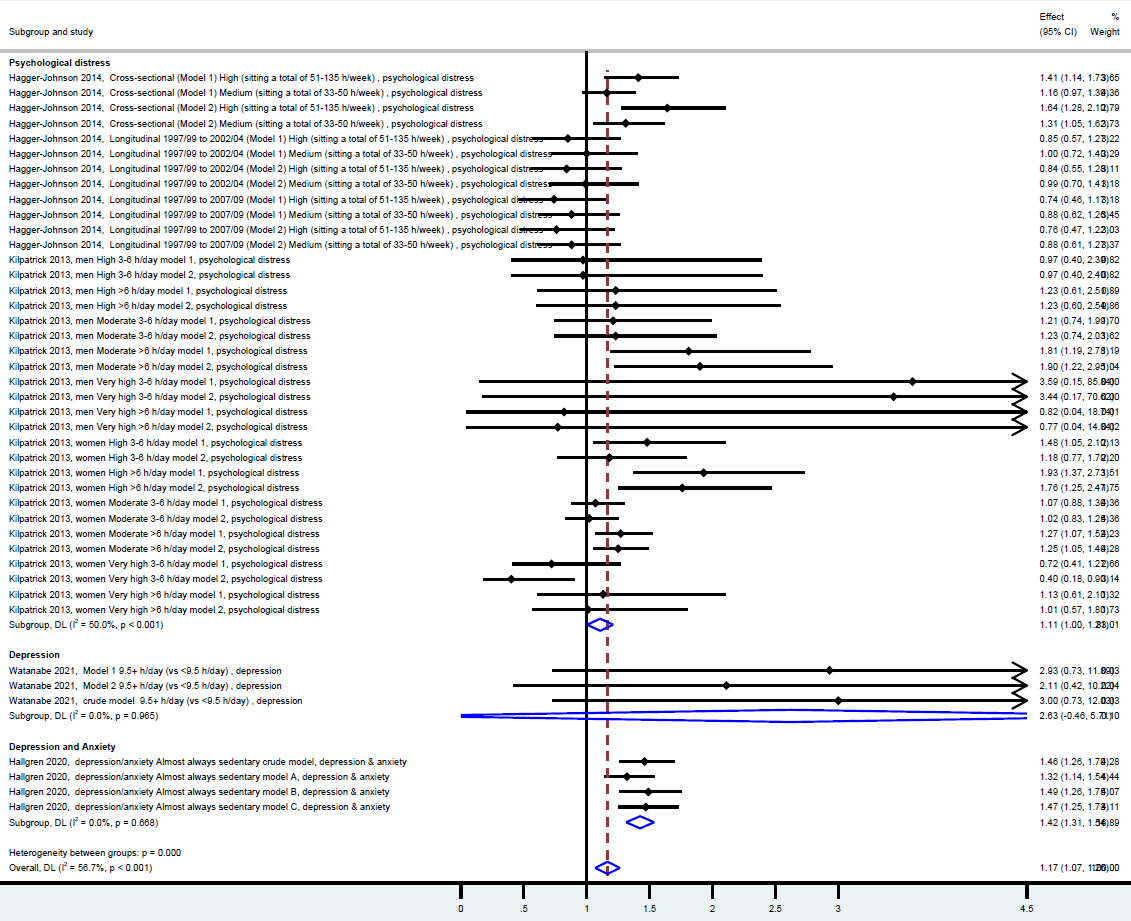


Risk of severe mental health issues

**(**only four studies assessing occupational sitting time**)**


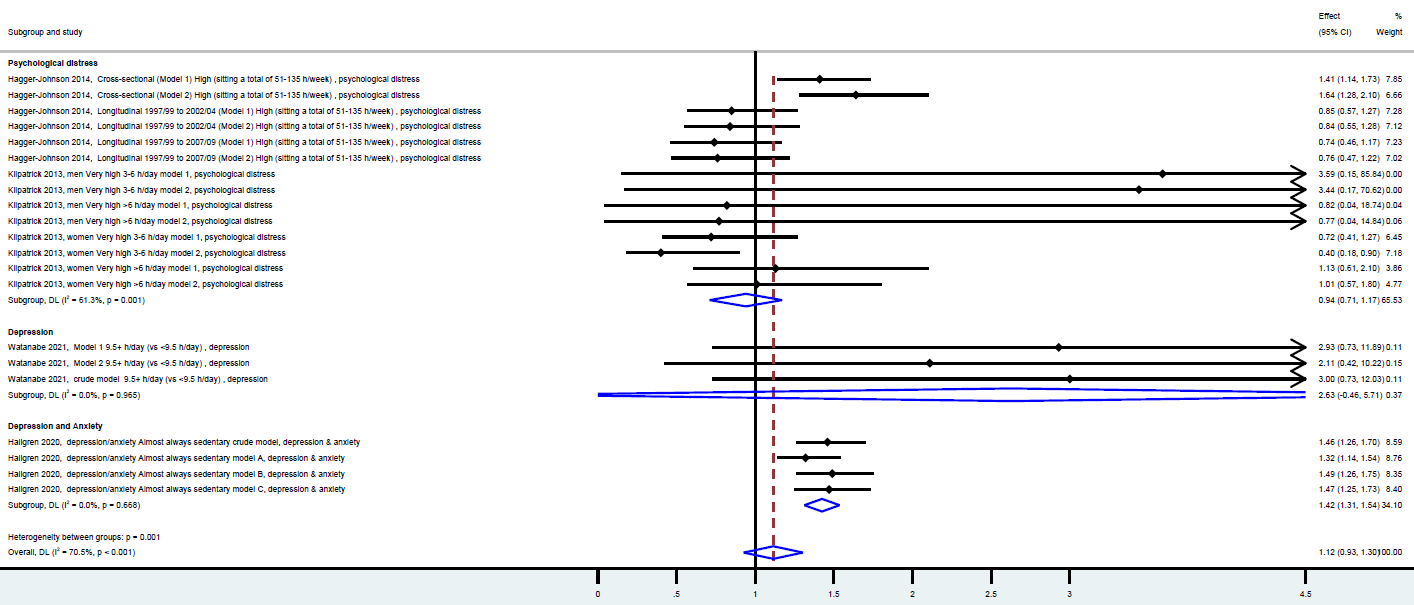


Risk of severe mental health issues

(Crude or less adjusted odds ratio - Pessimistic model)

**(**only four studies assessing occupational sitting time**)**


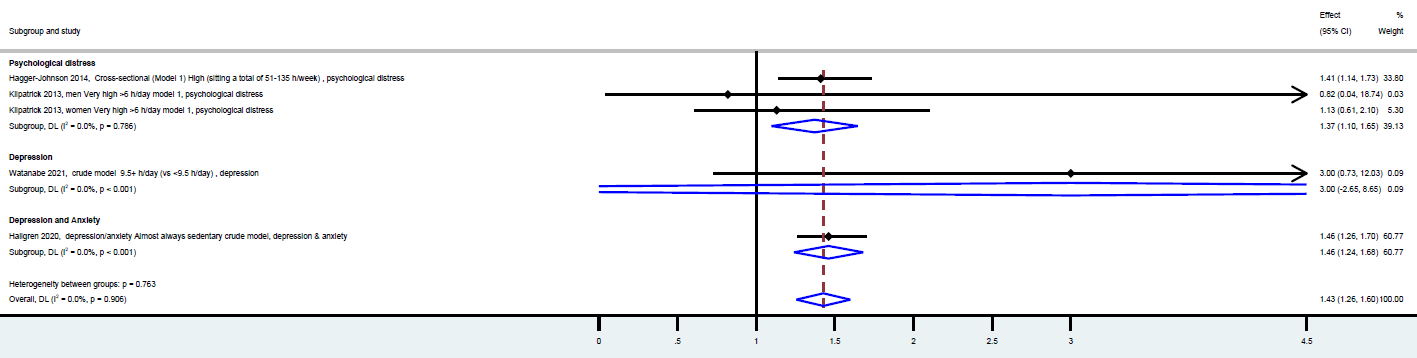


Risk of severe mental health issues

(Fully adjusted odds ratio - Pessimistic model)

**(**only four studies assessing occupational sitting time**)**


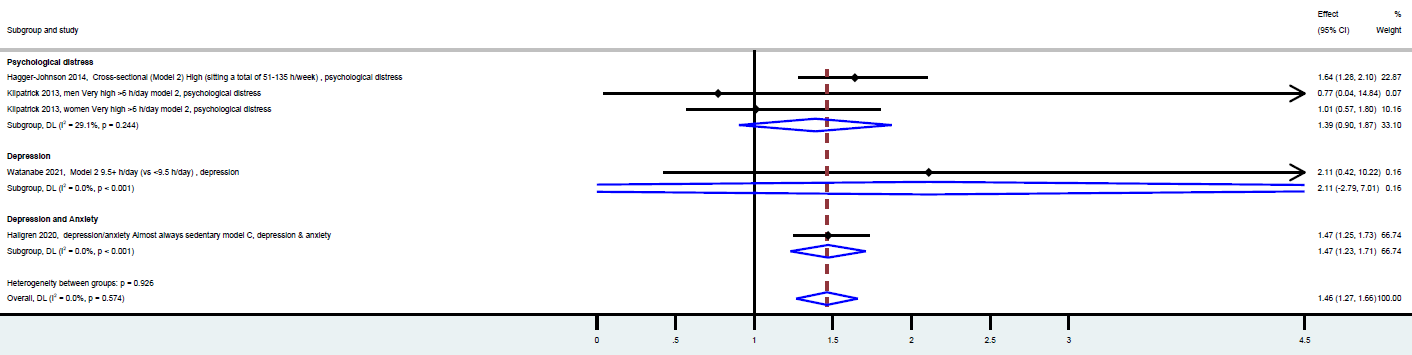


Risk of severe mental health issues

(Fully adjusted odds ratio – Optimistic model)

**(**only four studies assessing occupational sitting time**)**


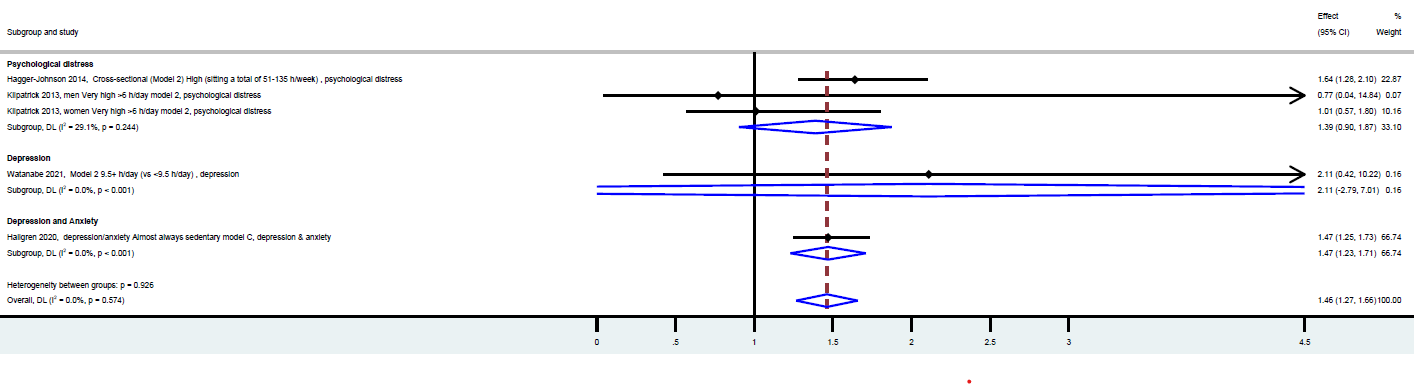


Risk of severe mental health issues

(Crude or less adjusted odds ratio - Optimistic model)

**(**only four studies assessing occupational sitting time**)**


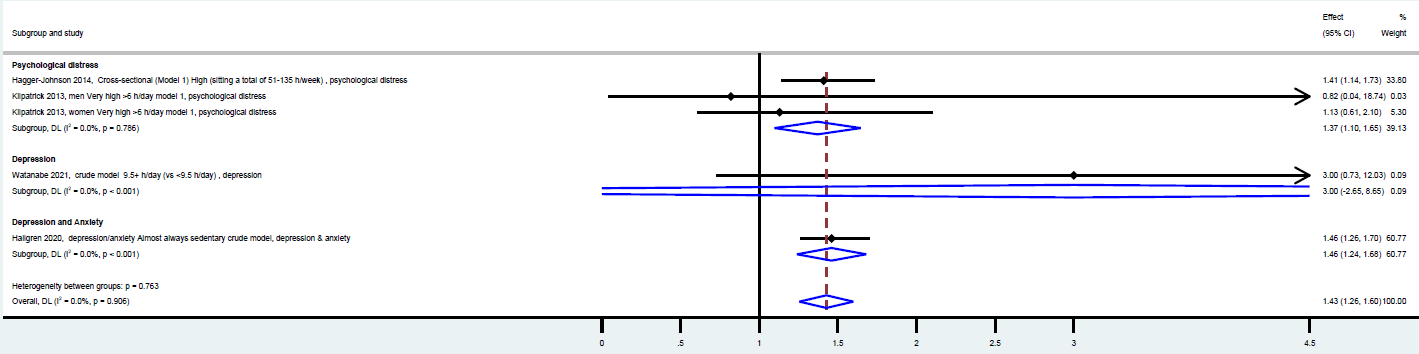

Supplement: S1 Fig — Blue lozenges represent the overall risk for each type of mental health issues after exposure to sedentary behavior. Dots represent the risk of mental health issues for each included study. The length of each horizontal line around the dots/lozenges represents their 95% confidence interval (95 CI). The black solid vertical line represents the null risk of mental health (with a value of 1). When horizontal lines cross the null vertical line, the risk of mental health is not significant. I-squared (%): percentage of heterogeneity between studies for each meta-analysis; Weight (%): Weight of each study for each meta-analysis. (DOCX) [file pone.0328678.s005.docx]
