# Supplementary material for: Impact of occupational sedentary behavior on mental health: A systematic review and meta-analysis
Source: PLoS One. 2025 Aug 20;20(8):e0328678. doi: 10.1371/journal.pone.0328678 (PMC12367128; doi:10.1371/journal.pone.0328678)

**Supporting Information**

# S6 Fig. Metafunnels for the risk of mental health issues due to occupational sedentary behaviour, depending on groups: 1) risk of mental health (both intermediate and severe), 2) risk of severe mental health (all risks), 3) risk of severe mental health pessimistic model (fully adjusted risks), 4) risk of severe mental health pessimistic model (crude or less adjusted risks), 5) risk of severe mental health optimistic model (fully adjusted risks) and 6) risk of severe mental health optimistic model (crude or less adjusted risks)

*Each dot represents a single study, with its corresponding effect size (x axis) and its associated standard error of the effect estimate (y-axis). Large high-powered studies are placed towards the top, and smaller low-powered studies towards the bottom. The plot should ideally resemble a pyramid or inverted funnel, with scatter due to sampling variation. Studies outside funnel plot are likely to present bias* ^62^*.*

Risk of mental health issues

(both intermediate and severe)


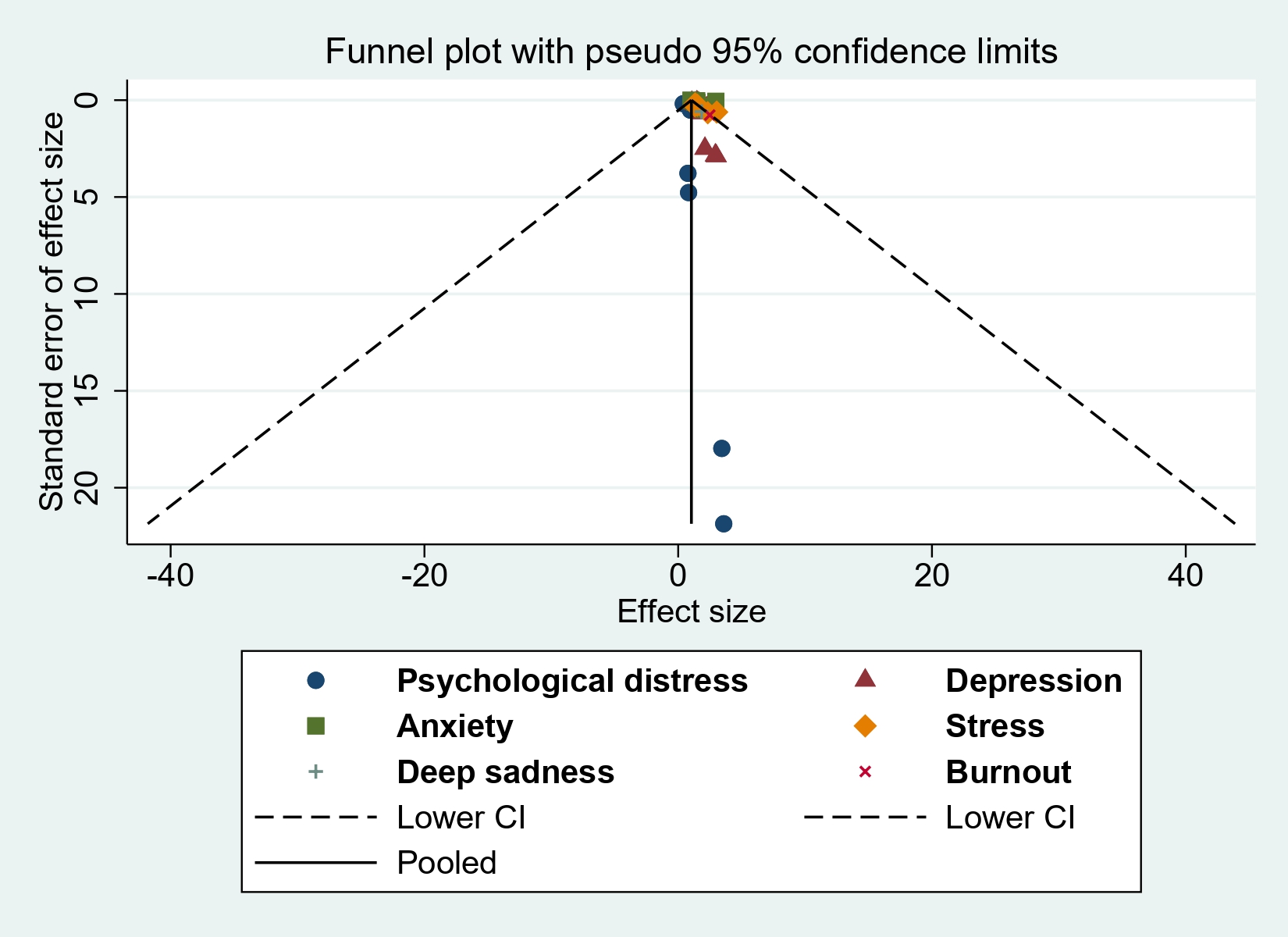


Risk of severe mental health issues

(all risks)


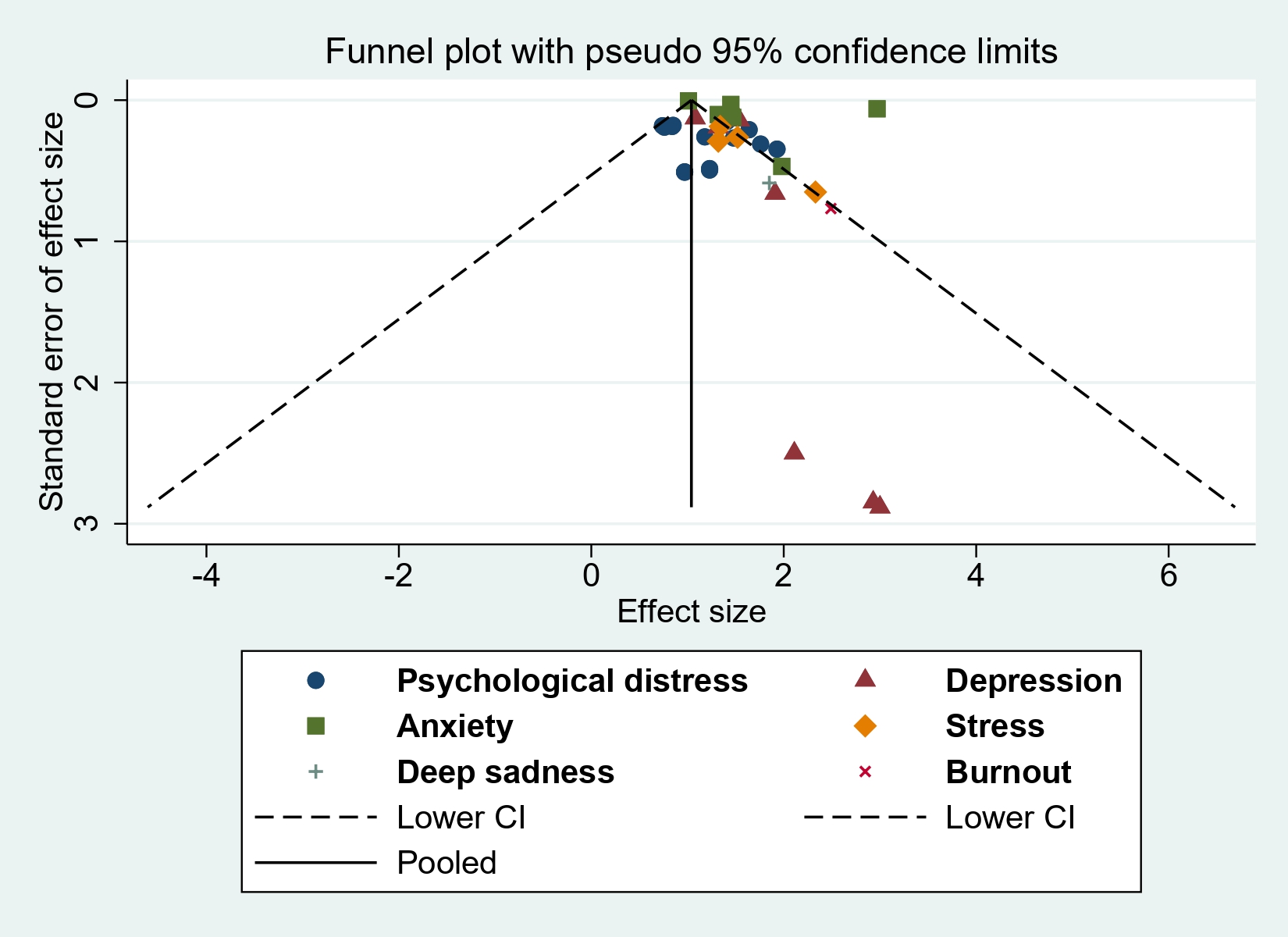


Risk of severe mental health issues

(Pessimistic models)

**Fully adjusted risks**

**Crude or less adjusted risks**


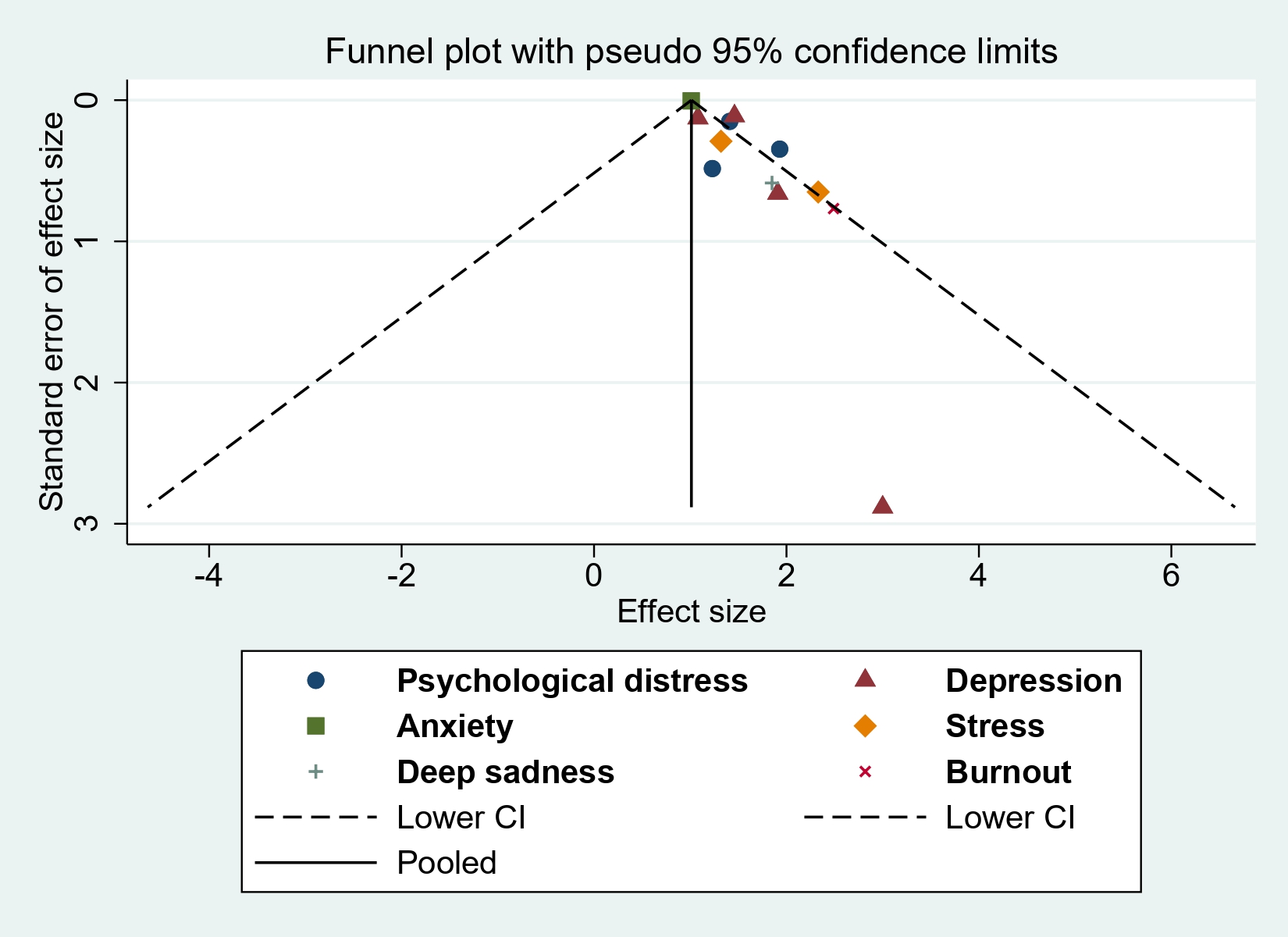

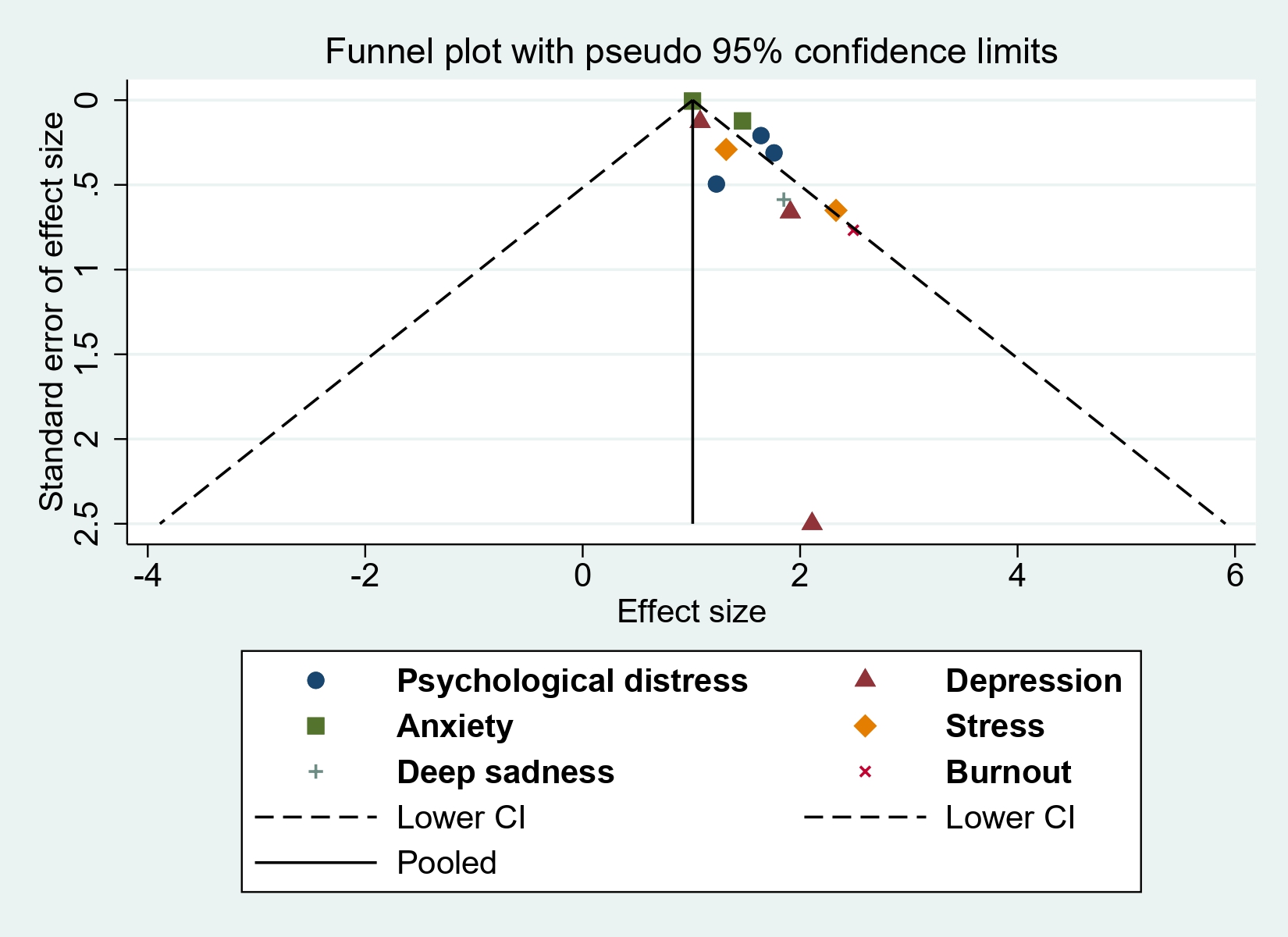


Risk of severe mental health issues

(Optimistic models)

**Fully adjusted risks**

**Crude or less adjusted risks**


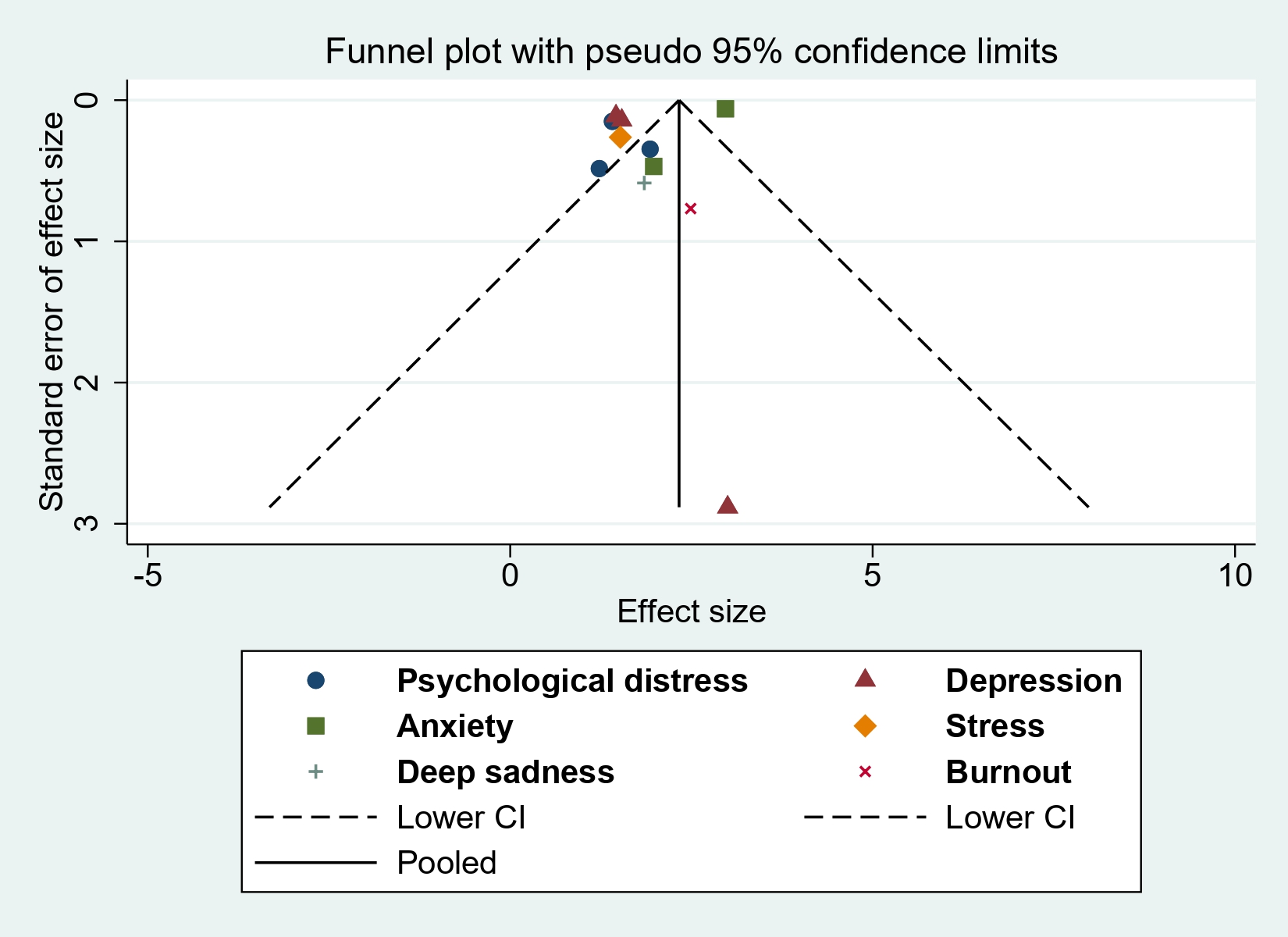

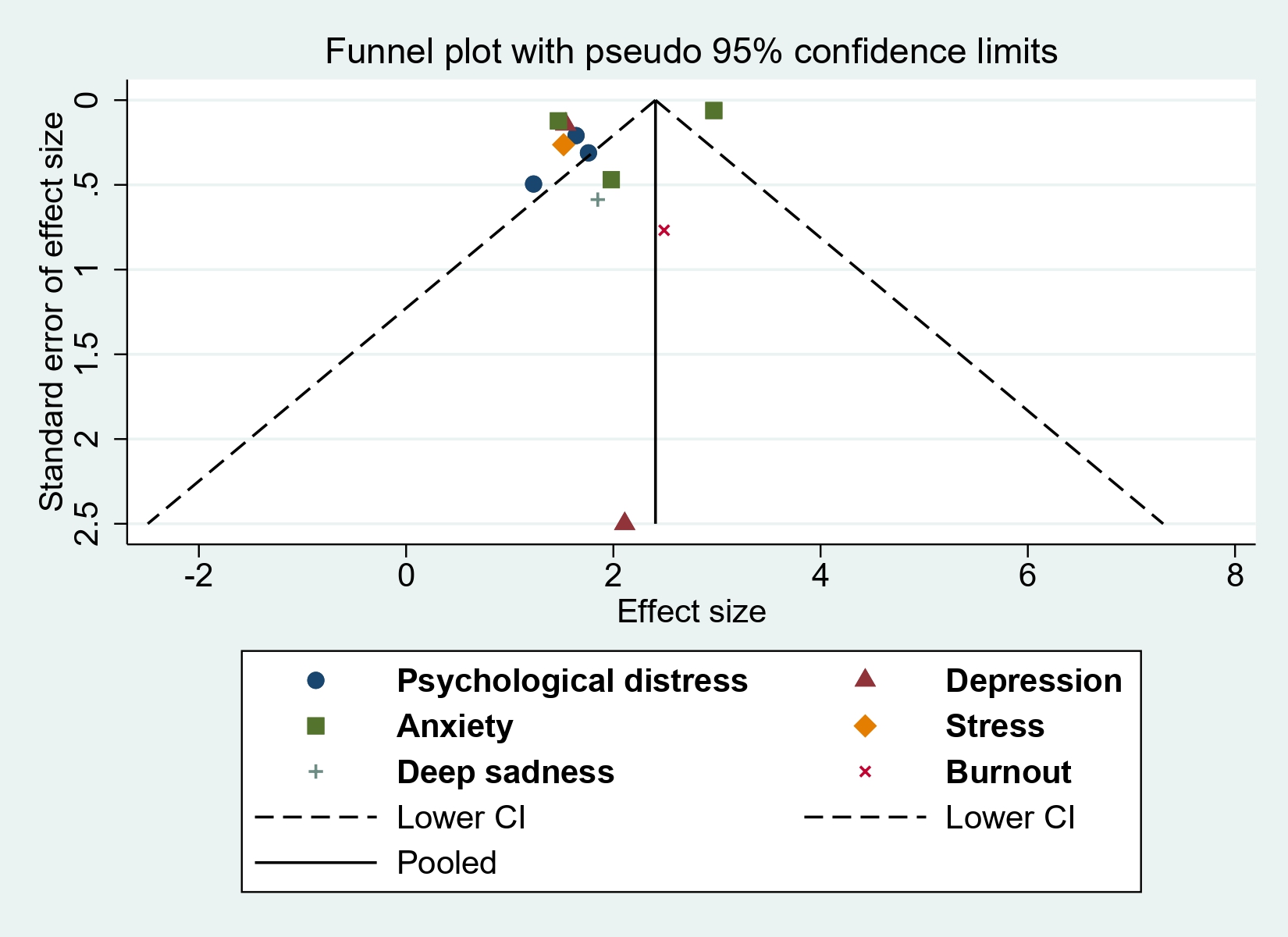

Supplement: S2 Fig — Each dot represents a single study, with its corresponding effect size (x axis) and its associated standard error of the effect estimate (y-axis). Large high-powered studies are placed towards the top, and smaller low-powered studies towards the bottom. The plot should ideally resemble a pyramid or inverted funnel, with scatter due to sampling variation. Studies outside funnel plot are likely to present bias. (DOCX) [file pone.0328678.s006.docx]
